# Entropy and Cities: A Bibliographic Analysis towards More Circular and Sustainable Urban Environments

**DOI:** 10.3390/e25030532

**Published:** 2023-03-19

**Authors:** Daniel R. Rondinel-Oviedo, Naomi Keena

**Affiliations:** Peter Guo-Hua Fu School of Architecture, Faculty of Engineering, McGill University, Montreal, QC H3A 0C2, Canada

**Keywords:** entropy, sustainable cities, circular economy, thermodynamics, urban systems, urban entropy, sustainable developing, urban studies

## Abstract

Cities are critical to a sustainable future for our planet; still, the construction and operation of cities rely on intensive resource and energy use and transformation, leading to the generation of waste, effluents, and pollution, representing negative externalities outside and inside the city. Within every process, transformation implies the use of energy and the increase of entropy. In an urban system, the transformation of energy and materials will trigger the creation of entropic landscapes, mainly in the informal city and in unguarded natural landscapes, even hundreds of kilometers away, which generates substantial economic, social, and environmental impacts. In this sense, cities are significant contributors to the environmental crisis. Upstream, degradation of landscapes and ecosystems is frequent. Cities’ externalities and exogenous consumptions are directly linked with entropy and entropic landscapes, which are recognized as pollution (in the air, water, and land) or waste and in the degradation of natural ecosystems and communities. Through a systematic review of existing literature, this paper first outlines briefly how entropy has been applied in different disciplines and then focuses on presenting recent developments of how entropy has been defined, used, and characterized in urban studies concerning sustainability in cities and architecture, and presents a definition of the concept in relation to urban systems and key aspects to consider.

## 1. Introduction

Cities depend on unsustainable linear flows [1,2,3] that leave behind pollution, desolation, environmental and social losses, and other problems at the beginning and end of each flow (water, material, energy) [4]. One of the main factors responsible for these negative impacts is the lack of understanding (or awareness) of entropy inside and outside the city and in the two edges of the materials and energy flows (upstream and downstream). Consequently, understanding entropy in the urban context will promote circular flows, better and more efficient use of the limited available resources, and design more sustainable cities.

However, the way entropy is framed in literature makes the concept alien and not easy to approach for architects, urban designers, and cities’ policy makers. The concept is perceived as negative [5] or intangible and related to information or statistics [6,7,8], and not to environmental effects in a specific territory. In addition, it is easy to discourage its study in urban sustainability due to the complexity and the copious and heterogeneous use in several disciplines [9]. Finally, the concept of entropy is not constantly or explicitly presented in the urban impact evaluation methods. Even though a lot has been written about entropy, there is still not a unique understanding of its pertinence as a tool to contribute to urban sustainability. Hence, a clear and straightforward approach and definition in relation to urban impacts are needed to start viewing entropy as a prospective element to address circular city models and sustainability. Some aspects that need to be considered are the contextualization of the concept in relation to non-equilibrium systems, irreversibility, and considering the arrow of time in every urban process [10,11,12], which entails positive and negative aspects, inside and outside the urban boundary.

By using a systematic literature review [13,14,15] and a bibliometric [16] and bibliographic analysis of the existing database, the research aims first to find and present how the concept of entropy has been utilized in urban studies in relation to the sustainability of cities and second, develop a framework where the concept of entropy is understood in relation to urban systems to open the possibility of defining how entropy can be used as a tool to design more circular and sustainable cities. Then, the research will address the question of how entropy has been defined and used in urban studies and which aspects from the literature should be considered and evaluated in the design of sustainable cities. Then, understanding entropy in the context of urban systems can help to design a comprehensive framework that contemplates the relationship between built and natural environments and informs future decision-making toward more sustainable urban environments with planned entropy. Finally, the research proposes a novel definition of entropy concerning urban studies and establishes the foundation for continuing the study of entropy as a lever for sustainable cities and landscapes.

Accordingly, this paper is structured in five sections. First, Section 1 is presented prior to the systematic review and describes a broad definition of entropy to narrow down and define the concept’s scope of use. Then, Section 2 describes the research strategy and methodology for the literature review; Section 3 shows the result of the systematic literature review. Finally, Section 4 and Section 5 are the Discussion and the Conclusions

### 1.1. Entropy Concept in Other Disciplines

Entropy is a broad concept that has undergone progressive development. It was used primarily in the nonsocial sciences, and then its use extended to other disciplines, including the urban and regional system studies [17]. The word was coined (in 1868) by the German physicist Rudolf Clausius. With its Greek prefix en-, meaning “within”, and the trop- root here meaning “change”, entropy basically means “change within (a closed system)”. The closed system we usually think of when speaking of entropy (especially if we are not physicists) is the entire universe [18]. From the definition of the word, the change or transformation process is an important aspect of entropy. In this original definition, the transformation is referring to energy (from useful energy to not useful). Many years before, Sadi Carnot, in 1827, experimented with entropy principles [19]. The Second Law of thermodynamics or the law of Entropy brought a new perspective to the mechanistic dogma of classical physics, where irreversibility was part of nature. Biological, social, or economic processes are governed by the Law of Entropy, and not by the laws of mechanics and “all systems, whether isolated or not, are subject to the same forces of entropic decay (…) any complex differentiated system has a natural tendency to erode, dissipate, and unravel” [20]. The notion of entropy has subsequently been adapted to other areas of knowledge, including thermodynamics (equilibrium and non-equilibrium), information theory, economics, art, ecology, and biology.

In thermodynamics, entropy measures a system’s disorder or randomness. For example, organized, usable energy has low entropy, whereas disorganized entropy, such as heat, has high [21]. Therefore, entropy is a measure of the complexity of the molecular states that depend on the heat content. If no heat is present (absolute zero), the molecules fall in simple arrangements such as crystal, and entropy is zero [1]. Energy is the critical concept in the First Law of Thermodynamics and entropy in the Second Law. Moreover, entropy is the only concept in the physical sciences having directionality with time, which means that applying the entropy concept to biology would lead to a deeper understanding of living systems [22]. However, the thermodynamic study of real systems is still an active work in progress and lacks an established corpus [9]. Therefore, analyzing cities from an entropy viewpoint implies understanding cities as biological-living entities, including time directionality.

Entropy from a thermodynamic perspective was later redeveloped as uncertainty, with the principle of entropy maximization applied as a statistical inference method for supporting spatial location and spatial interaction models [7]. In information theory, the concept flourished with the remarkable contribution of Claude Shannon, who gave a statistical character to entropy in his renowned paper “A mathematical theory of Communication” [6], a foundational theory for modern digital communications. Here, uncertainty is a synonym of entropy, and entropy is related not to energy but bits (short for binary digit) or information [7]. The “Shannon’s Entropy”, which measures the amount of information held in data (high entropy means spread out data, while low entropy means concentrated data) [8], has a substantial influence in many other disciplines, and it is used consistently and frequently in urban studies that are more related with spatial distribution, statistics, and information availability in an urban concentration.

In economics, scholars highlighted that economic development depends on the use (and degradation) of limited natural resources. Nicholas Georgescu-Roegen mentioned that “a living organism does not need just energy but low entropy which it sucks from the environment and degrades into high entropy (waste)” [23], in other words, Georgescu-Roegen argues that the function of the economy is primarily the transformation of “low entropy” into “high entropy” as cited in [24]. In parallel, Jeremy Rifkin highlighted that cities’ main problems have a common origin: “the massive energy inputs required to sustain contemporary city life, the entropy of the urban environment is rising dramatically to the point where the continued existence of urbanization is being called into question.” [19], hence, the idea of cities as entropy producers is essential for this research.

Regarding arts, Arnheim Rudolf refers to entropy with the idea of a clash of orders, where disorder is not the absence of all order but rather the clash of uncoordinated orders. He adds that any progress requires a change of order; for example, a revolution must aim at the destruction of the given order and will succeed only by asserting an order of its own [25]. Moreover, Robert Smithson developed arts interventions by reinterpreting the concept of entropic landscape, emphasizing the opposite relationship between waste and luxury, stating that “if we want a bigger and better car, we are going to have bigger and better waste.” He mentions that economics seems to be designed to exclude the entire entropic or remove it from the visual field of study. The entropic landscape that architecture generates is seldom considered, and how landscapes look after humans have exploited them is often overlooked [26]. However, the entropy that architecture and cities generate affects much more than the appearance, such as polluted and discarded landscapes and even the destruction and displacement of entire communities.

Finally, regarding ecology and biology, entropy is used from a different perspective. In this perspective, life processes degrade input resources to create new information, build an organization, add structure, or upgrade energy. The resources, once degraded, will become available again for new life cycles, driven by the entropic degradation of solar energy. In addition, the higher the ecological diversity, the lower the production of entropy per unit of biomass, because resources are better utilized and support the growth of the whole spectrum of ecosystem hierarch [27]. More related with Shannon’s entropy, Ramon Margalef introduced ecologists to the entropic method, and later he reflected the significance of entropy for different aspects of life: energy, succession, diversity, dissipative/self-organizing systems, creative constraints, and evolution [28].

### 1.2. Ideas from Other Disciplines Relevant for Entropy in Urban Studies

From all these areas of knowledge, the use of entropy within a broad biological perspective is central for this literature review since the approach to the city will be a systemic approach; even more, the city will be understood as a living organism. What is central to underline is that biological organisms are open thermodynamical systems exchanging energy and matter with the ecosystem and that after entropy, order, even life could occur. The evolution and growth of biological systems cannot be explained by the Second Law of thermodynamics of an isolated system but with the open system perspective, where an organism requires an exchange of matter and energy with their environment [29]. The chemistry Nobel prize-winner Ilya Prigogine is an outstanding figure for this approach, developing a thermodynamic study of real systems.

Central concepts that could be adapted to the urban perspective of entropy were developed and postulated by Prigogine, including complex system, self-organization, equilibrium, the arrow of time, and especially his theory of far-from-equilibrium systems, irreversibility, and dissipative structures. This is especially true for urban systems, which are located inside a natural ecosystem taking and dissipating energy (Fernández-Galiano, 2000). For Prigogine, entropy is much more than merely a disorder and its irreversibility, it is a mechanism for producing order and could be directed to a new organism and system [10], creating highly ordered complex structures with extraordinary properties, and create life itself [11], in his words, “the irreversibility of time is the mechanism that brings order out of chaos.” Finally, it is central to mention that according to Prigogine entropy principle applies to energy and materials “that matter is subject to an ‘irrevocable dissipation’ and that irreversibility is also a feature of material transformations.

## 2. Systematic Review Research Methodology

Entropy research is a developing subject in different disciplines, and in recent years, the concept was adapted to urban studies with different perspectives. To identify and describe current practices and incorporate front-line knowledge, a systematic literature review was chosen as a method of research, integrating findings and perspectives in relation to entropy from many studies [13]. The systematic review included three different stages and a previous phase. The phase before the systematic literature review, crucial for the next stages, was a study of the entropy concept in various disciplines. Then, the first stage was to establish the literature review’s scope, the study necessity, and the research question. The second stage deals with the definition of precise keywords to perform the academic search and with developing a research strategy, including preliminary searches, establishing the eligibility criteria and the definition of the data collection. Finally, the third and final stage was to evaluate the quality of the collected data, process, and present the results.

### 2.1. Scope, Necessity, and Research Question

After the general study of the entropy concept in other disciplines (Section 2), it can be argued that although the literature includes the use of entropy in urban studies, it is necessary to perform a comprehensive review to analyze how much we know and need to know about the role of entropy in urban sustainability. Consequently, the research objective is to find and present the recent developments of the entropy concept and its uses in urban studies to support sustainability and as a secondary objective, present how the concept was defined and characterized within urban studies. Hence, the research question is, how has the entropy concept been used in urban studies in relation to cities’ sustainability?

### 2.2. Keyword Definition, Bibliometric Analysis, and Research Strategy

The definition of the search strategy for the systematic review includes specifying the sources for consultation, the keywords, the filter criteria, the number of search rounds, and the exclusion standards. The search was performed using three prestigious academic search engines (Scopus, Science Direct, and Taylor & Francis). For the key words’ definition, three steps were applied. The first step was to define two main keywords used for this research. The first word was entropy and to find a second word, five searches in SCOPUS were conducted (Table 1). According to results, it could be seen that there is a gap between entropy and specific areas of research as “built environment” and “urbanism”. The combination with more results was <“entropy” AND “urban”>, which obtained 1001 documents and was selected for a preliminary bibliometric network analysis using VOSviewer version 1.6.18 [30] software, where the number of lines and the distance between concepts in the graphic show the interconnection between nodes and concepts.

**Figure 1:**
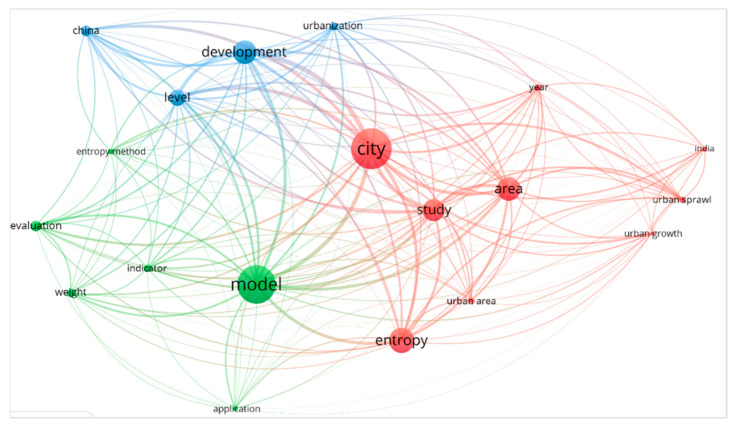
Preliminary bibliometric network analysis in VOS viewer, at 25 occurrences for “entropy” AND “urban” results in Scopus.

The first bibliometric analysis was performed three times, changing the number of occurrences (25, 50, or 100). The code for the software was: (Map created based on text data (RIS file) > Title and abstract > Ignoring structured abstract labels and copyright > Full counting > 20998 terms in total > (25/50/100) occurrences: (215/76/31) words > 60% > (129/46/19) words included) (Figure 1). We can determine that the words “entropy” and “urban” have an established correlation in the literature. Many resulting words are linked to “evaluation”; “measurement”; “indicators”; “dimensions”; “impacts”; and “problem”. Hence, “modeling” and “evaluation” are words linked to “entropy”. According to the word’s connection, “modeling” could be related to the evaluation of “urban growth”, “urban sprawl”, “urban area”. Another significant conclusion is that “China” and “India” are recurring words, which indicates that multiple studies have been performed in those regions. Finally, it is central to mention that the words “flow”, “waste”, “dissipative”, “pollution”, “circular”, “output”, and “sustainability” are not present in results, but they are present in “sustainable development”.

Since there is a high occurrence of words “measure” and “model”, they will be included in the final search. In contrast, some missing keywords essential for the present literature review are “flows”, “system”, “sustainability” (or all its variables), “circularity”, and “metabolism”. These words will also be included using the Boolean AND to reduce the search scope and be more precise. Finally, the second main keyword should be “urban”, but since “cities” also presented a high number of results, it will be used [“urban” OR “cities”]. With all these insights from the first search, the next step was to execute several preliminary searches in Scopus using a combination of identified keywords (Table 2), aiming to recognize the best word combination according to the number and content of the result.

From Table 2, in search #1A, from a total of 68,902 documents, 19.8% are from Physics and Astronomy, from Engineering 18.2%, from Computer Science 13.2%, from Mathematics 13.1%, from Materials sciences 9.4%, from Chemistry 5.9%, Chemical Engineering 2.7%, Biochemistry 2.4%, Earth and Planetary Sciences 2.1%, Energy 1.8%, and from other subjects 11.3%. Regarding the country of origin for #1A, China accounts for 29%; the United States for 20%; India 6%; Germany and the United Kingdom 5% each; Japan, France, and Italy 3% each; Canada, Russia, Spain, and Iran 3%; followed by another 139 countries with a smaller percentage. From searches #5 to #17, a different combination of keywords was used using the results from the preliminary bibliometric analysis.

Search #8 included the results from the preliminary search (i.e., have “measure” or “model”). Originally, 1071 papers were obtained, which was considered high for a systematic and detailed literature review. Then, filters were applied, including the exclusion by area and language, bringing a still high number of results (659). Later, titles were scanned, and it could be inferred that most of the results were not related to the research topic. Therefore, two actions were carried out: first, the exclusion of the words (“measure” or “model”) since they were leading to broad results and to results out of the scope of the research question studies. Second, the search strategy needs to be narrowed down. Subsequently, the next step was to use two levels of keywords for search. In the first level, the main words “entropy” and (“urban” or “city”) should be present in the title or keywords. The second level, keywords “flow” or “system” AND “sustainable” or “metabolism” or “circular” or “dissipative” should be present in the abstract or the title or the keywords. This coding was used in Scopus (239 documents) and adapted to Science Direct (92 documents) and Taylor and Francis (14 documents).

To compare the results from the original search (Table 2, #1A) with the new search code, the 239 results from Scopus were analyzed concerning the subject of knowledge and the country of origin. The results indicate that 26.7% of the documents are from Environmental Sciences, 14% from Social Sciences, 12.5% from Engineering, 8.8% from Energy, 6% from Earth and Planetary Sciences, 5.3% from Agricultural and Biological Sciences, 4.1% from Computer Sciences, 4.1% from Medicine, 3.5% from Biochemistry, Genetics, and Molecular Biology, 2.7% from Business, and 12.3% from other subjects. Regarding the country of origin, China accounts for 70%; the United States for 7%; Italy for 6%; the United Kingdom for 3%; India, Germany, France, Iran, Japan, and Taiwan for 2% each; followed by other 28 countries with 1% or less. From this last analysis, it is essential to emphasize the contrast in the disciplines compared to the initial results. Moreover, the preponderance of research from China is much higher with the new search code.

**Figure 2:**
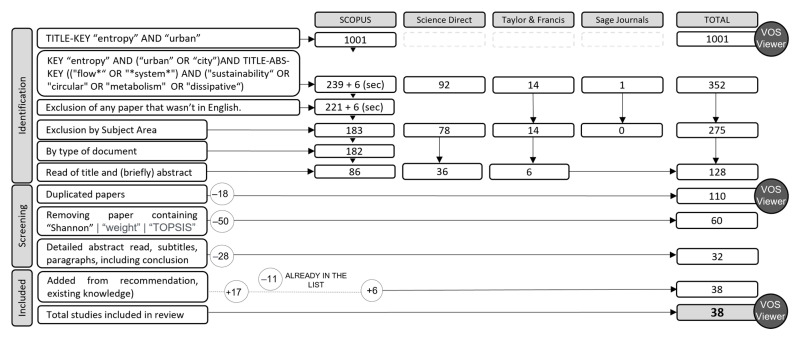
Systematic literature review filters, exclusion criteria, and number of papers per stage.

The next step was to apply distinct layers of filters (Figure 2). First filter language (English); second, related to areas of knowledge, filtering those not related with the research question; the third filter was reading all titles and scanning all abstracts for excluding all papers not related to the research question. From these filters, the number was reduced to 86 papers in Scopus, 36 in Science Direct, and 6 in Taylor and Francis. The following step was to join the results from the three databases in Zotero reference manager software, in order to produce one RIS file to be analyzed in VOS Viewer version 1.6.18.

**Figure 3:**
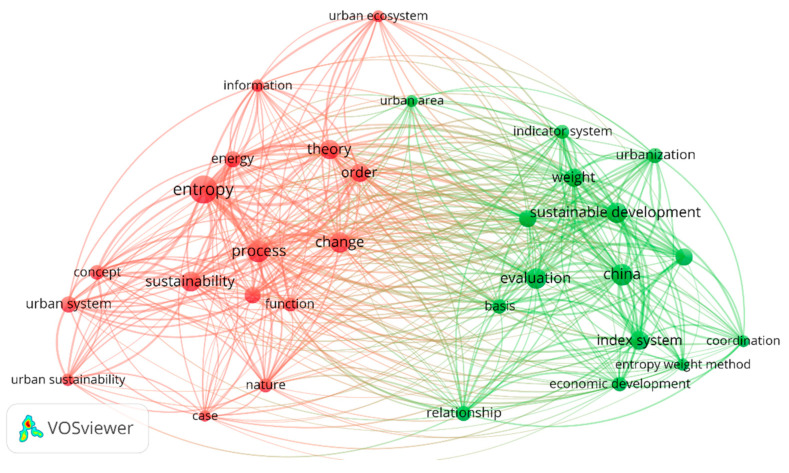
Second bibliometric network analysis in VOS viewer for 10 occurrences for Zotero RIS file (110 papers) as an example. Route: Map created based on text data (co-occurrence) > Read data from reference manager files [RIS] > Tittle and abstract (Ignoring structured abstract labels and copyright) > Binary counting > 3212 terms in total > at 10 occurrences: 51 words meets the threshold > 60% > 31 words most relevant are included.

In Zotero, the repeated papers were excluded, obtaining 110 papers. Then, a second bibliometric network analysis using VOSviewer version 1.6.18 was performed to evaluate the relevance of the results (Figure 3). For this second analysis, a binary counting was chosen, resulting in more specific results and the words being more logically linked. Three analyses were performed (15/10/3 occurrences). For 15 occurrences and 10 occurrences, two main clusters are present with similar characteristics. Cluster one deals with “entropy” related to theory (“concept”, “energy”, “theory”, “order”, “nature”) and its “application” in “urban systems” (“case”, “process”, “function”) for “sustainability”. Cluster two deals with “entropy weight method” and includes words such as “index system”, “evaluation”, “basis”, “weight”, “indicator system” in relation with the urban area as cases studies. When the search is broadened to three occurrences, the number of clusters arises to eight, and they are not clearly limited or separated. One insight is that “low entropy city” is a tangential word located in a tangential cluster. This cluster contains essential words for the research as “thermodynamics”, “waste”, “open system”, or “second law”.

Even using a narrow search code (Table 2, #17), the results include papers related to the “entropy weight method”. Even though this is a method that could be used to measure entropy in urban contexts by using statistical methods, this is not directly linked with the research question. In consequence, an additional exclusion criterion was implemented. Hence, documents that define entropy from the information theory and statistics, i.e., that include in titles or keywords the words “Shannon”; “information entropy”; “weight”; “TOPSIS”; or “fuzzy”, were excluded. Finally, the last filter was to perform a detailed read (abstract, subtitles, some paragraphs, conclusion) of the 60 papers and exclude all those where the use of entropy is not related with the research question. A total of 28 papers were excluded and 6 additional papers from author existing knowledge were added, resulting in a final count of 38 papers.

**Figure 4:**
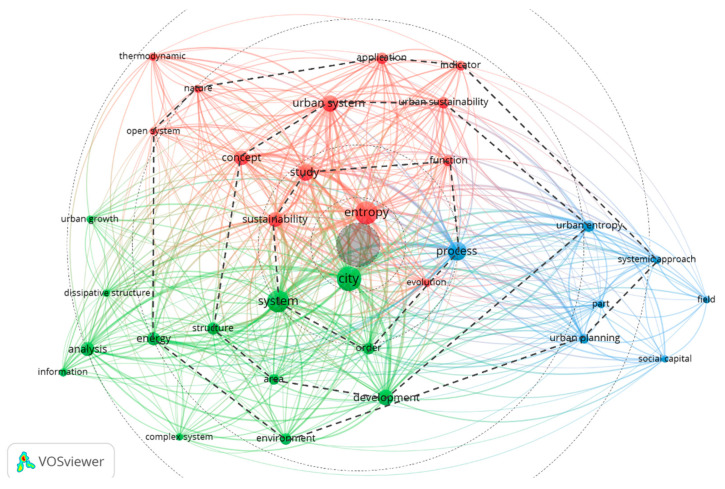
Final bibliometric network analysis in VOS viewer version 1.6.18 —Zotero RIS file (38 papers) route: Map created based on text data (co-occurrence) > Read data from reference manager files [RIS] > Tittle and abstract (Ignoring structured abstract labels and copyright) > Binary counting > 1131 terms in total > at 4 occurrences: 40 words meets the threshold > 100% > 40 words are included. Note that some words were excluded such as paper, approach, attention, assumption, or regard. Segmented lines and radial circles from the center of the network were added to understand the relation between the different topics and their relevance to the main area of exploration of this research which is the gray circle in the center of the image, between “entropy” and “city”.

In the last step, a final bibliometric network analysis using VOS viewer was made at 9, 6, and 4 occurrences to identify the main themes for review. From the four occurrences analysis, some conclusions could be presented. First, “city” and “entropy” appeared in the center of the diagram, closely connected with the system and process. Second, the present literature review research aim is between city and entropy. Using this area as the center, we can draw layers of closely related words and observe three clusters. The red cluster is linked to the urban from a sustainable perspective and includes urban sustainability, urban system, open system, or thermodynamics concepts. The second cluster (green) relates to the city’s systemic vision from a territorial approach and spatial analysis. It includes central words such as “structure”, “area”, “order”, or “development”, and “urban growth” or “complex system” that is eccentrically located. Finally, the third cluster (blue), is linked to processes and planning. A relevant word in this cluster is urban entropy, close to the systemic approach (Figure 4).

For the content analysis of this research, three themes linked to the previously identified clusters were defined. First, the definition of entropy in relation to sustainability. Second, the connection between entropy and the vision of the city as a system, including identification of analyzed flows (energy, material, air, water, carbon). Third, the process or measure method (and instruments). An additional theme is related to the solution/ideas for circularity from each paper.

## 3. Results of Literature Review

The literature review results are presented in three sections. The first is a descriptive analysis of the results in terms of year of publication, area of knowledge, and country of origin. The next section is a content analysis focusing on three main topics: entropy definition, entropy in urban context, and entropy characterization. The final section is a table with a summary of the 38 reviewed papers.

### 3.1. Descriptive Analysis

**Figure 5:**
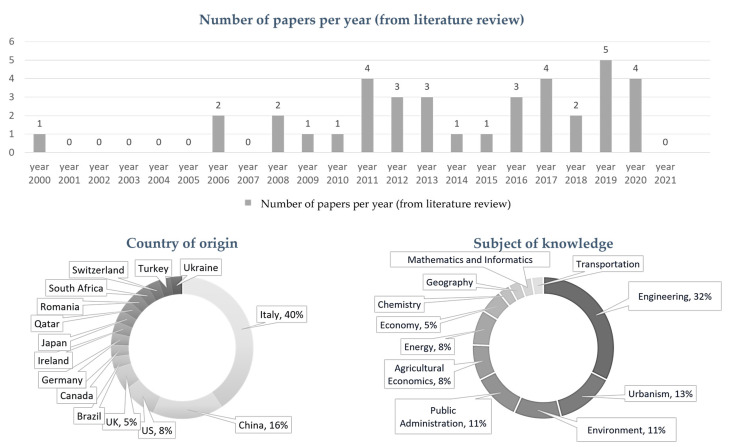
Analysis of year of publication, the subject of knowledge and the country of origin of final selected papers.

The 38 resulting papers were processed to analyze the year of publication, the subject of knowledge, and the country of origin (Figure 5). First, the bibliographic information was transcribed manually to an Excel spreadsheet for the descriptive analysis. Then, each subject of analysis (i.e., country, area of knowledge, or publication year) was counted. The results vary considerably compared with results from the first searches (Section 3.2). A key aspect to mention is that the main subject of knowledge is engineering (32%) and that urbanism is the second (13%), which is more in the area of interest of present research. Moreover, it is essential to mention that the corresponding author’s affiliation was considered to determine the area of knowledge. An important footnote to this point is that a brief verification of the second author’s affiliation was also performed, noting that 19% of the papers have an author with an architecture background. Regarding the country of origin, Italy accounts for 40%; China for 16%; and the United States for 8%, followed by 12 other countries. The contrast in the disciplines is evident when compared with previous results. Finally, regarding the year of publication, the oldest paper is from 2000, and the most recent one is from 2020.

### 3.2. Content Analysis

The next section is a content analysis focusing on three main topics: entropy definition, entropy in urban context, and entropy characterization.

#### 3.2.1. Definition of Entropy in Relation to Urban Studies

**Figure 6:**
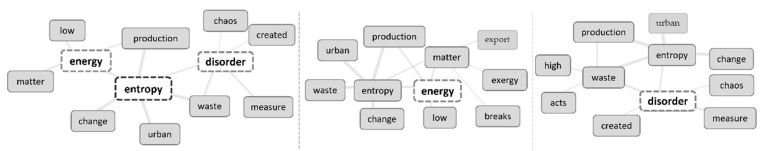
Entropy two main connotations: energy change and order change. Graphic based on definitions from 38 papers and elaborated using Voyant tools software, [31].

In the revised papers, entropy is related to change with two main connotations, the first is related to change in energy and the second to change in order (Figure 6). Regarding the energy implication, this definition is aligned with the classical description of thermodynamics, where entropy is associated with the change in the energy production of a system, hence, matter and exergy are the two associated key concepts. One finding is there is a weak understanding of the relation between energy and matter in most of the papers that uses this approach. However, the relation between energy and matter is central to connect the concept of entropy to urban studies. One concept that helps in this sense is the dissipative structures’ definition. Concerning entropy as disorder, this approach is related with the production of waste and the creation of chaos. Waste is described as high entropy material and in general terms, entropy has a negative connotation and is described as the opposite of sustainability, from pollution to social conflicts. Note that these two connotations (energy and order) could be related if order is defined in relation to energy, which could be the case to define entropy from urban systems.

##### Internal and External Entropy

**Figure 7:**
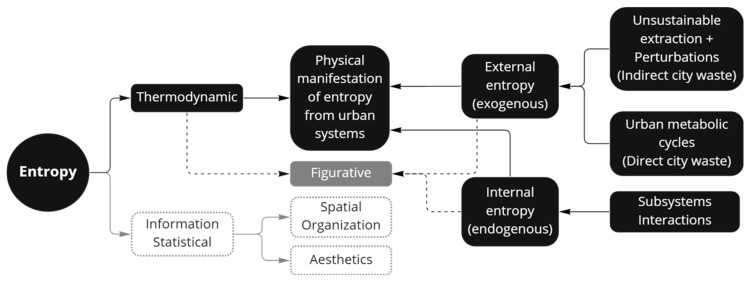
Entropy classification in relation to type and place of production. For present research, the effort is to focus on the thermodynamic definition of entropy. The figurative entropy concept relates urban environmental impacts to entropy, but from an abstract perspective, i.e., it is not directly related to thermodynamics concepts and calculations are not established equations or methods yet. Based on a combined interpretation from [9,35,37,38,39].

One basic aspect to address is the classification of entropy in terms of where **it** is produced in relation to the urban system. A common categorization throughout the studies is the internal and the external entropy (Figure 7). These categories are associated to the conception of cities as open and non-equilibrium systems, where at least two system are interacting and interchanging matter and energy, and entropy is produced inside or outside the system boundary. The urban ordered structures were created increasing disorder outside the system [29,32]. Rees and Wackernagel stated that an open self-organized system needs to import available energy and material from their host environments in order to not run down, as cited in [33]. The internal entropy, also named endogenous, is related with interactions of subsystems, while external or exogenous relates to direct city waste (from metabolic cycles) and indirect city waste (produced by unsustainable extraction in source ecosystems) [34]. Another related definition of internal entropy is the presence of nature in former industrial landscapes; despite not being referred to directly as internal entropy, it can be linked to urban entropy inside the city [35]. Entropy in urban systems was defined as the sum of external entropy fluxes and internal entropy production [36].

##### Entropy and Urban Systems Energy Dispersion

**Figure 8:**
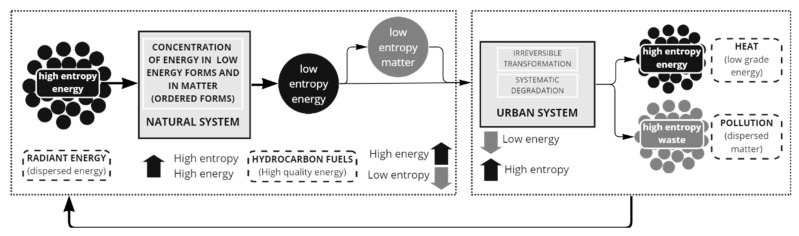
Entropy and energy relation in an open system, from natural to urban system.

Energy is another key aspect to address, closely connected to the relation of internal and external entropy since a decrease in available energy in a system is related with the increase in disorder. Later, the maintenance of the internal non-equilibrium steady state of cities requires continuous energy draining from an external source. Entropy is produced when energy is used, and the more energy a city uses, the more entropy it will produce, and cities use massive amounts of energy to grow [29]. Note that in this case, it is important to relate energy also as the embodied energy from materials. Hence, entropy is also presented as an indirect impact of energy use, which produces an irreversible entropic flow, more specifically, as sinks for unused anergy [40]. In complement with this idea, entropy could be also characterized as a measure of the dispersion of energy, thus, radiant energy arriving from the Sun is transformed causing countless entropy-producing flows, and urban systems usually require the use of low-entropy sources of energy (as fossil-fuels). The energy ultimately leaves as high-entropy heat waste that radiates back into space, but also as waste or pollution, that ends up dispersed in natural systems (Figure 8). This process of energy transformation is related with the notion of the city as a dissipative structure and complementing the idea from Prigogine of “order out of chaos” the city will produce “order out of (additional) chaos” which is produced in external and internal systems.

An additional aspect regarding energy is that from the classical view, or even more, a purist view of the subject (the one linked to the thermodynamics of Clausius), the production of entropy should be directly related to energy flows and cannot be expressed in terms of material waste or pollution; in this sense, the entropy that returns to the natural environment could only be waste heat, since “thermodynamics does not include the notion of usefulness or utility, and because of this, failure to capture the human degradation of resources” [24]. Hence, a new conceptual frame is needed to address the notion of utility and therefore, the irreversibility of a process.

##### Entropy, Irreversibility, and Dispersion in Natural and Urban Systems

About the relation between irreversibility, utility, and entropy, some authors argue that only irreversible processes produce entropy [36] while other authors link the irreversibility with the concept of utility loss, arguing that the more irreversible a process is, the more potential utility is lost. Hence, entropy could measure the irreversibility of the transformation inside a system [41] or the development capacity, which quantifies the potential that a system has for development and it could be the proxy for entropy [42]. In the same direction, irreversibility is present when energy is transformed. Sustainability will be obtained if the irreversible entropy flux of human activities is lower than the negative entropy flux from the sun [40]. A final annotation regarding energy and entropy is that in papers that use equations for entropy calculation, the unit that is used to express the specific entropy of a system (S) is the kilojoule per kilogram per kelvin (S = (kJ/kg·K)).

Finally, in relation to change in order, in the revised studies, there are two main approaches. The first is related mainly with spatial arrangement, measuring the level of dispersion of an urban area, that is translated then into an indicator of risk in urban systems [7]. Within this line, entropy is related with dispersed a morphology as urban sprawl or scattered urban configuration. The second approach is related with the information availability for a system [43] or the level of dispersion of the information, including statistical information to calculate the weight of an index [44] or to project social interactions [45].

#### 3.2.2. Entropy and Urban Studies

As other authors noted, entropy within urban studies is linked to two different approaches: the first approach is connected with information theory and regularly employs the formula developed by Shannon. Here, entropy is usually a measure related to information (spatial) and aesthetics; the second approach is related to thermodynamic laws with a tangible and scientific nature; it could be called physical entropy and is related to energy, waste, and heat or pollution that the cities generate. One of the biggest challenges within urban studies is to relate these two different approaches [35]. For the first approach, the statistical information definition of entropy, there is abundant literature concerning applying this type of entropy within urban studies. This approach to entropy does not deal directly with sustainability and the physical impact of urban systems. However, it is important to acknowledge its relevance and enormous presence in urban studies and to identify aspects that usually are interested in spatial distribution, statistical information, and some social aspects that could be measured with metrics from central agencies. Concerning the second approach, the energy use in an urban area is responsible for the order of a city but also is responsible for the disorder in the environment. Hence, the biosphere urban planners should employ free forms of energy, following the low-entropy concept [38]. This idea is palpable when referring to energy, which could be directly measured, but it is less evident when referring to materials, air, or water.

##### The City as an Open System and as a Dissipative Structure

Connected with the thermodynamic approach, a critical aspect concerning entropy and urban systems is the vision of the city as an open system. This vision includes the definition of the boundary, the identification of the flows (energy, material, air, water) and the effects on the urban and natural systems. In this vision, it is central to recognize three main points. First, the city-ordered structure is possible due to the generation of more significant disorder in the environment out of the open system. Second, the city absorbs external input (negentropy) and emits internal output (entropy, heat). Third, the non-equilibrium steady state of the city should not be confused with thermodynamic equilibrium [36]. Most of the authors are using the vision of the city as an open system as a general context of their research, arguing that cities are complex dynamic entities, open systems constantly importing energy and matter across their boundaries [46]; that cities are open, complex system which processes material flows as inputs and discharges waste outputs [33]; or that cities “need high-value energy (with a high exergy component, e.g., oil, gas) and release unsustainable, scarcely reusable, low-value energy (entropy), which often constitutes an environmental problem and a cause of ecosystem alteration” [38].

However, entropy flow is not always presented as a negative aspect; it could also represent the harmonious capacity of systems, and the entropy production could reflect the ability for system reduction and reproduction in the metabolic process and can indicate the vitality of the system [47]. In complement with this low entropy, this state is ideal in transportation, utilities, services, and in the arrangement of built-up areas, “however, some redundancy and diversity (entropy) in the urban area is required so that the city can better resist long-tail events likely to compromise its functioning (natural catastrophes, man-made events)” [7].

**Figure 9:**
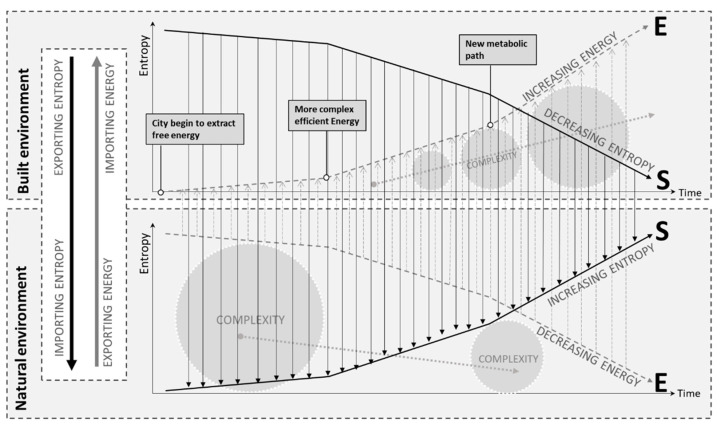
City as a dissipative system and the entropy budget of Urban System and the Natural Environment. Evolution towards more complexity inside the city will increase entropy (disorder) in the natural environment. Some positive feedback from the urban system to the natural environment or a circular approach inside the urban system will be needed at some point to avoid collapse. An increase in entropy leads to a decrease in available energy. Energy is also related to the embodied energy of materials and services. In an urban system, the lack of energy input will result in an increase in entropy. Adapted from [29,48].

**Figure 10:**
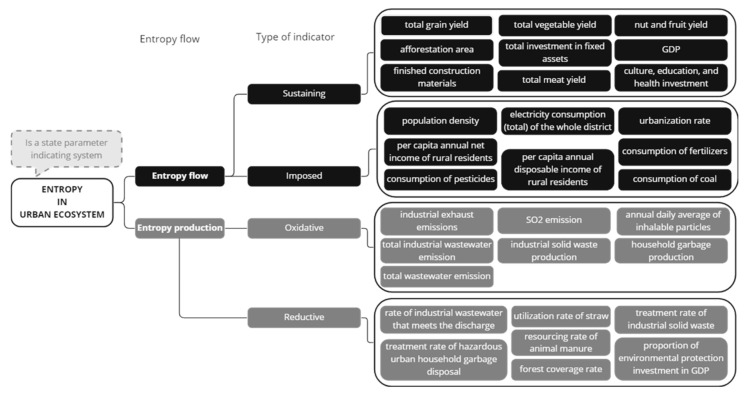
Proposal of entropy assessment indicator for an urban ecosystem. Adapted from (Xuan et al., 2012) [47].

Similarly significant, the concept of dissipative structures (Figure 9) was constantly referred to in analyzed papers and is fundamental for the thermodynamic approach to entropy within urban systems. The dissipative structure theory is related to material flows, where “low-entropy materials enter, undergo a series of changes in their energy and entropy state and, after a time lag, the residual high-entropy materials or wastes are dissipated or returned to the environment” [33]. The principle of dissipative structure states that urban ecosystems are a “typical dissipative structure deviating from equilibrium, so its entropy flow and entropy production have different meanings from the traditional ones” [47] and aims to describe the timeline dynamics, some terminologies as oxidation or reduction have been applied for evaluating urban ecosystems (Figure 10). The dissipative structure theory was also related to energy dissipation. Energy is dissipated as heat; however, the amount of energy dissipated will be in opposite relation to the maturity of a system, which could mean that a mature city has a lower dissipation rate than a new one [29]. Finally, the dissipation of materials is not clearly described in the literature, and again, it should be developed for a comprehensive analysis of entropy in urban systems.

Finally, some other aspects of the urban system and entropy addressed by revised papers are that entropy addresses three main features in the urban system: the position or location, the mechanic flow network, and the system scaling or size [7]. These three main features should be contrasted with the two main approaches (physical and statistical).

#### 3.2.3. Characterization, Measure Method, and Instruments for Entropy in the Literature

**Figure 11:**
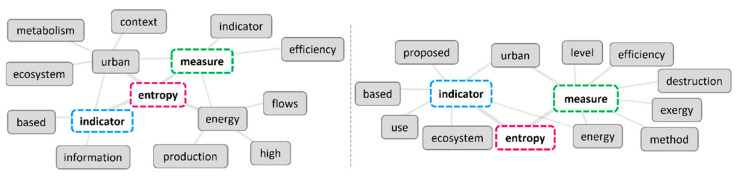
Concept links for characterization, measure method, and instruments. Based on annotations from the author about characterization, measure method, and instruments from 38 papers using Voyant tools software version 1.6.18 [31].

No single practice was found or a method that is repeated in two or more papers. Despite general equations from open system theory being referred to in documents related to the non-equilibrium system, it was more to establish a broad context (city as an open system). There are some coincidences in general perspectives regarding characterization, such as the approach (thermodynamic, figurative, or information/statistical) or some other similitudes, such as recognizing that there is an external and an internal entropy. Still, no common base was founded for the proposed elements, indicators, proxies or indexes for entropy. In parallel, it was noted that indicators were more related to urban systems, and measuring is more related to energy and efficiency (Figure 11).

The entropy measurement method for open systems continuously refers to the Prigogine equations for open systems, where the entropy is the sum of the negative entropy produced inside the system with the positive entropy created in the environment. Here, entropy change depends on the interactions among constituents of the system and exchanges with the external environment. The equations presented in the analyzed papers are summarized in Table 3. In relation to these equations, some authors have developed a method to measure the entropy production as a proxy for measuring actual consumption and the irreversibility transformation of the resource consumption by using LCA tools [41]. However, this method was not applied to urban studies. As a complement, an exhaustive review of thermodynamics in ecology can be reviewed in [49].

##### Entropy Is a Measure of Energy Efficiency

Another aspect that is frequently measured and related to entropy is energy. For instance, an entropy indicator was presented as the ratio between the entropy variation due to energy losses of buildings and the entropy variation due to the solar energy gain of a mesh area [40]. In another study, entropy has been described as a characteristic of energy, where high entropy is related to solar energy (also called soft energy) and low entropy is referred to as fossil or nuclear energy (also named hard energy) [50]. In addition, entropy production has been presented as closely related to the destruction of exergy. That is to say, entropy production and exergy destruction measure how efficiently energy is used. Hence entropy is a measure of energy efficiency [24]. The same author argues that from a thermodynamic perspective, entropy should be measurable, at least in theory. “To be of any use as an indicator, one must be able to measure thermodynamic entropy in thermodynamic units of JK−1. Without a mathematical formulation, it remains a metaphor, and one which we argue fails to be useful…”. Thus, strictly talking about thermodynamics, the indicator should be measurable in terms of SI unit: joules per kelvin (J⋅K−1). One example referred to by [46] is the “entropy pump” hypothesis developed by Svirezhev (2000) who stated that the production of entropy by an ecosystem could be used as a metric for quantifying the anthropogenic stress on an ecosystem; however this method it has been not applied to urban systems. Hence, if matter, pollution, or noise cannot be measured in these units, then an alternative approach for entropy should be developed or adopted.

##### Entropy as Negative Impact

This is exemplified by some figurative strategies that have also been developed in the literature, aiming to measure entropy from another perspective (not in terms of joules per kelvin). For instance, some general strategies were presented by [5,43,51,52], where entropy is given as a measure of a negative impact or a dissipative material that becomes entropy, and in order to prevent these adverse effects, it is necessary to evaluate the internal entropy, in three central urban systems: the physical, the functional, and the social. A good illustration of this was given by the author using food waste (from crops, industry, and supermarkets), pointing to waste as “dissipated material” that becomes entropy, i.e., waste, which implies a further cost for its disposal, without considering the harmful effects on landfills or incineration systems. An alternative idea to evaluate entropy is to use the sustainability evaluation criteria of urban systems but in a negative way. For instance, the level of entropy could measure the unsustainability in the economic system [53], or the sustainability indicators could be the possible starting point in order to achieve the final target of this study: to assess urban entropy [37]. Another aspect that has been measured in relation to entropy is the efficiency in terms of metabolic processes, where with the intention to perform a material and product flow analysis and assess urban metabolic inefficiency, the following were quantified: (i) production, imports, and exports of raw materials; (ii) the manufactured good production and trade; and (iii) the waste production; and entropy is related to waste as high-entropy material [33].

##### Other Evaluation Methods and Indicators

**Figure 12:**
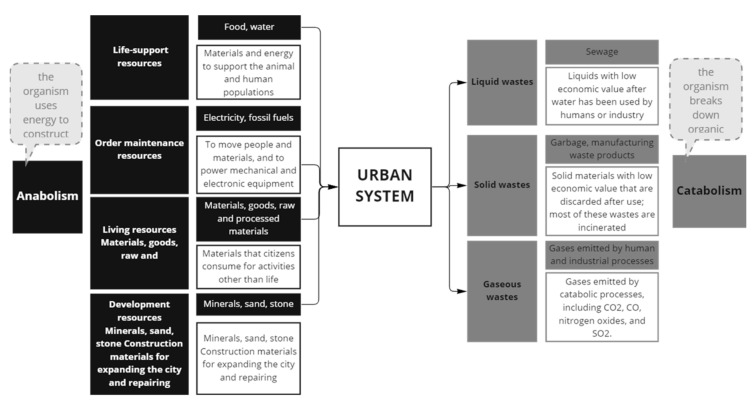
Metabolic elements in an urban system, some input resources have a low level of entropy (Living and Life Resources). The features in this graphic could be considered to evaluate entropy. Figure elaborated by the author adapted from [32].

Other diverse methods have been proposed to measure entropy, even if these methods do not measure entropy in thermodynamics units. For instance, [42] referred to Odum’s 24 attributes to describe ecosystem development. Specifically, it refers to entropy (attribute #23) and presented a method to calculate the system’s DC (develop capacity). From another perspective, [32] suggested several metrics to quantify metabolic activities and relationships in a dynamic and complex system. Some of these metrics could be related to entropy (Figure 12).

In contrast, a list of general entropy indicators based on the “low-entropy city principle” was developed. Some strategies are using renewable and local energies, local food chains, investment in public transport and smart mobility, and intelligent management systems for blocks of buildings, among others (Table 4). In the same line, the same author presented indicators to evaluate an urban system in terms of water quantity and quality parameters. These indicators have an explicit spatial character for practical decisions [34]. Another perspective is the top-bottom method using statistical data for five aspects of the urban system. Although this method is related to information/statistical entropy, it is pertinent to evaluate the proposed index and evaluation criteria for green development (Table 5). Finally, [44] presented an interesting perspective of using the evaluation of the “ecological services” as a reference for ecological load entropy evaluation, relating the concepts of heterogeneity and vulnerability. Hence, a system with a higher ecological load entropy has lower heterogeneity and is more robust and less vulnerable to disturbances.

### 3.3. Review of Central Aspects of Each Paper from the Literature Review

This section reviews the 38 papers in relation to entropy and its definition, the connection to urban studies, and the characterization and measure methods. Table S1 presents the list of these 38 papers, ordered by year (the table is presented as additional material). Furthermore, 11 additional documents that were not part of the search results and the bibliometric analysis were included at the bottom since they offer a different perspective of the entropy concept.

## 4. Discussion

Entropy is a broad concept that is still under development in many disciplines. It has been used in numerous fields, including thermodynamics, economics, statistics, communication, arts, and urban studies. For the present literature review, the focus was on the entropy that entails a physical manifestation in the natural or urban system. Hence, those papers related to statistics and information theory and not directly linked to a physical indicator of entropy are not part of this review. However, some exceptions were made to papers that passed through the search filter strategy. Those papers explore the connection between the statistical method and urban sustainability indicators.

### 4.1. A Growing Interest, a Change in the Perspective, and More Research in the Global South

The results from the descriptive analysis regarding the field of knowledge confirm that the three main areas are related to urban studies (engineering, urbanism, and environment), which infers a growing interest in this study of entropy within the field. In addition, many of the studies are based in Italy (40%), where entropy is more related to the perception of negative output and from a figurative approach to thermodynamics. It is also valuable to point out the minor presence of countries from the global south. Excluding China, 3 out of 38 are from that region (Brazil, Qatar, and South Africa), representing a gap in the literature and an opportunity to develop studies in those regions.

### 4.2. Three Key Considerations: The City as An Open System, the Location of Entropy Production, and That Entropy Production in Cities Is Unavoidable and Related to Energy and Matter Transformation

Three main aspects arise from the results concerning the definition of entropy. First is the idea of considering the city as an open system. Second, there are two main zones where entropy could be produced (inside and outside); note that the boundary is usually not considered. Third, any transformation process of any kind will require energy, and the use of energy entails entropy production. These three points are closely related since the exchange of energy (and matter) between systems (inside/outside) is associated with the conversion of energy (heat) and matter related. It is also important to point out that some of these aspects have been studied for decades (not necessarily with the thermodynamic-entropy approach). In an open system, the order of the internal system increases (due to exergy and matter consumption) and the entropy decreases (in a specific location and for a short time). Still, the overall net effect increases the disorder [54]. Cities “interchange different classes of inputs and outputs (matter, energy, and information) in the earth, the atmosphere, and the waters” [55]. To define precisely what is and what is not entropy is central to design tools that could measure it. For instance, internal entropy has been referred to as a physical manifestation in the form of urban decay landscapes [56], but it was also referred to as negative impacts of urban development as emissions (air, water, electromagnetic, noise) [5]. Therefore, it is essential to identify if the entropy is the cause or the effect and how to measure it.

One key aspect to fully incorporating entropy as a valuable tool towards sustainable cities is to clarify the relation between energy and matter concerning entropy. As previously reviewed, cities could be defined as a dissipative structure since they are open systems, far from the equilibrium state, with fluctuations and a nonlinear interaction mechanism [57]. In this context, the entropy concept should be used not only concerning energy but also to matter. Cities could be described as highly ordered dissipative structures. They take massive quantities of valuable energy and material from the ecosphere and then “dissipate” or export entropy as degraded waste or disorder to maintain their highly ordered dissipative structure [20].

However, this idea introduces some failures because entropy’s original conception was related to thermodynamics and linked to heat and energy. In the case of material waste or pollution, the link with energy is not clearly established yet, and the association with the classical concept of entropy is vague. Still, it could be possible that every system or material affected by urban development could be measured not as the quantity of energy used (exergy) but as the quantity of energy needed to restore the system’s original state. For instance, restoring a forest to its original condition before deforestation will require an enormous amount of energy (and time) to replenish the energy taken and transported to the city, not only for the wood but also for biodiversity lost. What is important to understand is the link between entropy and pollution, system degradation, or waste, considering that in systems (urban or natural), a decrease in energy is correlated with the increase in disorder, hence an increase in entropy. The biological system develops in the opposite direction of entropy, towards a lower entropy and away from equilibrium [58]. However, this evolution is only possible by increasing environmental entropy; “… we can clean up our pollution, but it takes work to do so” [59]. An urban system’s entropy could be assessed if we calculate the amount of energy required to reverse a process, clean the pollution, and convert it to an entropy value. Hence, the irreversibility (and utility) could be a pivotal aspect to address in the effort to link entropy and matter degradation.

### 4.3. Irreversibility Should Not Be Related Only to Negative Impacts

Concerning irreversibility, some initial concepts described as anti-entropic processes that “takes diffuse material and contrate them” were explained in contrast to the entropic process that takes concentrated materials and diffuses them through the ocean, the earth’s surface, or the atmosphere [55]. The dissipative structures and entropy have one intrinsic characteristic, which is irreversibility. Using irreversibility to produce structure is similar to the processes we see in a town, where there is some unavoidable pollution. Still, there are also some positive aspects as universities, lectures, cultural events, birthday celebrations, etc. You cannot have one without also having the other [10], in other words, irreversibility produces not only negative aspects. These positive aspects are rarely highlighted, and it is important to acknowledge them. “Systems that exchange entropy with their exterior do not simply increase the entropy of the exterior but may undergo dramatic spontaneous transformations to “self-organization” (…) from convection patterns in fluids to life [60]”.

Another concept related with irreversibility is the idea that matter is embodied energy [61], and is in continuous degradation depending on the constant input of energy and information to maintain its structure. For example, the erosion process as rust or mold on an abandoned building will appear sooner or later (if energy—or work—is not injected). In this case, irreversibility and utility is related with energy injection (and energy use could be related to entropy). Not related to urbanism, some recent ideas related to irreversibility have been developing, such as the Degradation-Entropy Generation (DEG theorem) that established a direct relation between material/system degradation and the irreversible entropies produced by the dissipative processes that drive the degradation [62] or the idea of use the entropy generation to measure “the degree of damage, the amount of the life of materials expended, and the extent of the life remaining” [63].

In the same line, one of the main problems of the use of entropy as an evaluation measure in urban development towards sustainability is that the original application of the concept needs to be related to energy. Even the initial approach of disorder or randomness resulted from the loss of energy. The energy here is required to maintain a system’s “ordered” state. Thus, if you inject more energy into a system, there is more chance that this system will be more “ordered”. Moreover, a system with more energy tends to function better, but this is not a straightforward law. For instance, the energy contained in a rainforest is potentially higher than in a desert, so the level of entropy of such a system is lower than in the desert.

On the other hand, when we remove a tree from the jungle, we move the energy contained in that tree from the forest to the city. Hence, we inject more “energy” or order in the city but create disorder or entropy in the forest. Moreover, in the case of the desert example, underground, we might find petroleum. However, this material is not part of the “dessert” ecosystem since it is inside the Earth’s crust, and from a sustainability perspective, it should remain there [64]. In any case, when the energy from petroleum is taken to maintain the order of the urban system, entropy is generated in the desert ecosystem. This entropy could be measured in terms of the disturbance of the affected natural ecosystem (the desert). As Constanza mentioned, another alternative to energy could quantify in monetary units, but this approach should be evaluated.

### 4.4. How to Measure Entropy from Urban Systems

One opportunity identified is the development of tools for measuring the entropy of the urban flows for each subsystem (energy, materials, water, air, and carbon). It is evident in the case of energy since entropy was initially formulated in that context. However, the entropy generated is still a blurry concept for water, air, or materials. Ideally, one single measure that includes time, area, and energy use should be used. For instance, the unit of measurement could be related to footprint; in this case, the entropy could be the piece of land, the energy, and the time needed to absorb the output flows or produce what is required to restore a system to the initial state. In this scenario, entropy measured could be the area of the territory affected by an urban activity in the function of the time it could take to regain equilibrium or initial state, or the amount of energy or work required to return to the initial state. In this case, entropy is the disturbance in ecosystem services and functions used [65], activities that undermine the functionality of these ecosystems and their capacity as an adequate ecosystem service. The description of the sustainability of a city should be measured not only in relation to the city itself but concerning the environments from where the city is importing negentropy and exporting entropy [66]. The sustainability of the natural environments that provide energy, water, and matter to cities should be considered when measuring cities’ sustainability.

## 5. Conclusions

The present research had the objective of finding and presenting how the concept of entropy has been utilized in urban studies concerning the sustainability of cities. Therefore, to reach this goal, first, a broad definition of entropy was presented, and second, a systematic literature review was conducted, analyzing 38 articles related to the research objective.

Entropy is a broad concept, and there is no established definition of the concept for urban studies. However, two main types of studies were identified, those that deal with the entropy from a thermodynamic perspective and those that understand entropy from a statistical/information perspective. The thermodynamic entropy approach is less developed and usually employs a figurative form. Even more, entropy related to energy (as its original definition) is rarely present in urban studies. It is generally unrelated to matter (and components) or too narrow when presented. In addition, a lack of a tool for visualizing urban entropy was observed. This is probably due to the absence of a unique definition.

One aspect that is always present is that the concept of entropy within urban studies should be contextualized in relation to non-equilibrium systems. The non-equilibrium thermodynamics is a developing science that is still evolving. However, this perspective allows a positive approach to the concept, where entropy could be related to creating life or new systems out of the chaos, not only to disorder or uncertainty. Within this perspective, irreversibility is a crucial concept to develop. The introduction of the arrow of time in the urban process is key to understanding the entropy in the urban systems and establishing that the irreversibility of a process entails positive and negative aspects. One of the main remaining challenges is identifying tools and methods to quantitatively measure the entropy of this open non-equilibrium urban system and link this to energy and matter. One of the key observations of this paper is precisely to highlight the dependence of cities on natural systems. Energy from natural systems is injected into urban systems in order to prolong the life of the city and prevent degradation. Understanding or being able to monitor or control the growing entropy can allow for more sustainable development to be tracked within the dynamics of urban systems.

Concerning the characterization process and the measuring method (and instruments), the most evident conclusion is that there is no established method or theory to measure and characterize the entropy within the urban system and specifically for the thermodynamic approach and does not have an established corpus.

Finally, a definition of entropy, considering the reviewed literature, the main current uses of the term and the origin of the word (en + “tropos” (the Greek word for transformation)), is proposed to develop further research about entropy in the urban context. Hence, the proposed definition is: “entropy is the number of irreversible changes introduced in nature by urban systems, each time an energy or material transformation is required by cities to continue operating”.

Entropy as part of urban system studies is a little explored topic, especially in the Global South. In this region, cities are projected to grow exponentially and will require enormous energy and matter transformation processes. Hence, vast quantities of entropy will be generated and physically manifested in the natural and urban systems. Accordingly, the study and understanding of entropy are vital. Part of the future work is precisely defining what part of this physical manifestation in the natural and urban systems is and is not entropy. A key aspect is to explore the city as a dissipative structure. In this context, the energy needed to restore a system’s original state before the transformation could be measured. Alternatively, the disturbance of the affected natural ecosystem could be measured. A central aspect to consider is that entropy effects are related to time, space, and matter. This should be considered when developing tools for measuring the entropy of urban flow subsystems, such as energy materials or water.

The remaining problem is identifying and measuring the irreversible changes upstream and downstream, in the past and the future, improving understanding of entropy within the urban system, and developing specific instruments, indicators, and methodologies to measure entropy inside and outside the cities. Then, entropy will help to identify and illustrate the parallel and correlated landscapes between a city and nature.

## Figures and Tables

**Table 1:** Initial search in Scopus to find a second main keyword for the research.

Search Words	Number of Documents Identified by Academic Search Engine (Results)		
	A. TITLE	B. TITLE-KEY	C. TITLE-ABS-KEY
First search			
“entropy” AND “urban”	225	1001	2441
2. “entropy” AND “cities”	109	467	2394
3. “entropy” AND “urban system”	11	29	75
4. “entropy” AND “built environment”	0	19	82
5. “entropy” AND “urbanism”	1	2	9

**Table 2:** Search strategy and number of outcomes from Scopus. The three additional words concepts) for search are: “flow” (3 to 12), “measure” (13 and 14), and “sustainability” (15 and 16). Search #17 shows the final combination for search, which was applied in the other search engines. Note that the asterisk symbol (*) was used as a wildcard to broaden the search and find words with the same initial letters, as in “urba*” means that the result could have the words urban or urbanism.

Searched Words in Scopus	Number of Document Results		
	(A). TITLE	(B). TITLE -KEY	(C). TITLE-ABS-KEY
“entropy”	68,802	179,451	286,996
2. “entrop*”	73,406	185,097	300,124
3. “entrop*” AND “urba*”	250	1095	2740
4. “entrop*” AND (“urba*” OR “cit*”)	354	1621	5202
5. “entrop*” AND (“urba*” OR “cit*”) AND “flow*”	5	53	399
6. “entrop*” AND (“urba*” OR “cit*”) AND (“flow*” OR “input” OR “out*”)	7	94	1541
7. “entrop*” AND (“urba*” OR “cit*”) AND “flow*” AND (“input” OR “output”)	0	0	42
8. “entrop*” AND (“urba*” OR “cit*”) AND (“flow*” OR “input” OR “out*”) AND (“model*” OR “measur*”))	1	36	1071
9. “entrop*” AND (“urba*” OR “cit*”) AND “flow*” AND (“model*” OR “measur*”))	1	19	302
10. “entrop*” AND (“urba*” OR “cit*”) AND (“flow*” OR “input” OR “out*”) AND “sustain*”	0	3	206
11. “entrop*” AND (“urba*” OR “cit*”) AND (“flow*” OR “input” OR “out*”) AND (“model*” OR “measur*”) AND “sustain*”	0	2	144
12. “entrop*” AND (“urba*” OR “cit*”) AND “flow*” AND (“input” OR “output”) AND “sustain*”	0	0	6
13. “entrop*” AND (“urba*” OR “cit*”) AND (“model*” OR “measur*”)	100	636	3452
14. “entrop*” AND (“urba*” OR “cit*”) AND (“model*” OR “measur*”) AND “sustain*”	6	71	495
15. “entrop*” AND (“urba*” OR “cit*”) AND “sustain*”	18	195	702
16. “entrop*” AND (“urba*” OR “cit*”) AND “sustain*” AND “flow”	2	2	44
17. (KEY (“entrop*” AND (“urba*” OR “cit*”)) OR TITLE (“entrop*” AND (“urba*” OR “cit*”)) AND TITLE-ABS-KEY ((“flow*” OR “*system*”) AND (“sustain*” OR “metabolism” OR “circular” OR “dissipat*”)))			239

**Table 3:** In an open system, entropy can be maintained at a steady-state dS = 0 by interchanging energy with the exterior, so dSe is a negative influx of entropy. Based on [29,36,41].

Formula	Legend
dS = diS + deS diS > 0	dS: increment in the entropy diS: entropy production (heat, disorder) caused by irreversible processes within the system deS: entropy of exchange processes between the system and its environment.
deS = −diS and dS = 0	

**Table 4:** Entropy indicators according to [30].

**Environmental, social and economic phenomena, low-entropy nature-based solutions and entropy indicators.**	**Entropy Indicator**	**Low Entropy Nature-Based Solutions**	**Internal entropy indicators**	**Ecological Complexity**	**Social Complexity**	**Structural/Physical Complexity**	**External entropy indicators**	**Biosphere/Regional Complexity**
	Urban heat island	green roofs, tree plantations, parks		n° of species, n° of trees, covered area	People and enterprises involved	Internal or local T variation		Urban heat island, energy cost, CO_2_ emission
	Urban storm water	SUDS		n° and typology of SUDS	People and water enterprises	% Infiltration or stored water		Outlet flooding, global runoff
	Urban storm water	phytoremediation plants, wetlands		n° and typology of species, local freshwater ecosystems health status	People and water enterprises	Re-used water		Health status of receiving freshwater and sea ecosystems, CO_2_ emission
	Biodiversity loss	Diffused green and blue areas		n° of species, n° of trees, covered area	n° and typology of visitors and residents	spatial distribution of land uses		Landscape connectivity
	Sedentary and stressful jobs, traffic, pollution	Blue and green areas		n° of species, n° of trees. covered area	n° and typology of visitors and residents	spatial distribution of land uses, local air quality variation		Extra-urban trips, CO_2_ emission, regional health system cost
	Economic crisis, aging of buildings	Blue and green areas		n° of species, n° of trees. covered area	n° and typology of residents and tourists, enterprises involved	spatial distribution of land uses		Geographic distribution of visitors
	Social degradation	Blue and green areas		n° of species, n° of trees. covered area	n° and typology of visitors and residents, n° of crimes	spatial distribution of land uses		Socio-ecological issues in countries and ecosystems related with unresponsive and unconscious city resource consumption
	Increased food necessity, overpopulation	Urban gardens		n° of species, n° of trees. covered area	People and enterprises involved	spatial distribution of urban gardens		Imported food, CO_2_ emission

**Table 5:** Index to evaluate green development using information entropy according to [44].

Construction of Green Development Evaluation Index System in Cities	
Criterion Layer	Index Layer
Enhancement of living environment	Per capita urban public green areas (m2), X1
	Percentage of greenery coverage in built-up area (%), X2
	Percentage of the number of days with good air quality (%), X3
	Percentage of surface water up to and above Class III (%), X4
	Percentage of surface water below water section Class V (%), X5
Treatment and utilization of pollutant	Treatment rate of urban sewage (%), X6
	Innocent treatment rate of urban garbage (%), X7
	Comprehensive utilization rate of industrial solid waste (%), X8
	Reusing rate of water for key industrial enterprises (%), X9
Improvement of ecological efficiency	Water consumption per unit of GDP (m3/104 RMB), X10
	Energy consumption per unit of GDP (TEC/104 RMB), X11
	COD emission intensity per unit of GDP (kg/104 RMB), X12
	Ammonia nitrogen emission intensity per unit of GDP (kg/104 RMB), X13
	SO2 emission intensity per unit of GDP (kg/104 RMB), X14
	NOx emission intensity per unit of GDP (kg/104 RMB), X15
Optimization of economic growth	Per capita GDP (104 RMB), X16
	Share of the added value of the tertiary industry accounted for GDP (%), X17
	Share of the added value of advanced manufacturing accounted for above-scale industrial (%), X18
	Share of the added value of high-tech manufacturing accounted for above-scale industrial (%), X19
Development of innovative potential	Share of R&D expenditure accounted for GDP (%), X20
	Percentage of R&D Personnel to Employed Persons (%), X21
	Share of Expenditure on R&D to Sales Revenue (%), X22
	Share of Expenditure on Sci-Tec to General Fiscal Expenditure (%), X23

## Data Availability

This is a literature review, no new data was created.

## Supplementary Materials

The following supporting information can be downloaded at: https://www.mdpi.com/article/10.3390/e25030532/s1, Table S1: Key contributions from 38 paper included in the literature review. References [67,68,69,70,71,72,73,74,75,76,77,78,79,80,81,82,83,84,85,86,87,88,89] are mentioned in the Supplementary Materials file.
