# Supplementary material for: Entropy and Cities: A Bibliographic Analysis towards More Circular and Sustainable Urban Environments
Source: Entropy (Basel). 2023 Mar 19;25(3):532. doi: 10.3390/e25030532 (PMC10048483; doi:10.3390/e25030532)
Supplement: Supplementary file 1 [file entropy-25-00532-s001.zip › entropy-2045397-supplementary.pdf]

entropy

MDPI

Additional material

Table S1 - **Key contributions from 38 paper included in the literature review**. The following table presents relevant ideas taken from the referenced articles and some annotations made by the authors, which are written in square brackets [ ].

Year/author	Entropy definition in relation to sustainability and circularity	Entropy & urban studies (city as a system). Flows analyzed [energy, material, air, water, carbon]	Entropy characterization, measure method & Instruments
(Balocco & Grazzini, 2000) [40]	Entropy is presented as an indirect impact of energy use. Sinks for unused energy, or anergy, which often translates into entropy. Energy use produces an irreversible entropic flow. Sustainability will be obtained if irreversible entropy flux die to human activities is lower than the negentropy flux from the sun.	Energy is evaluated to measure the sustainability of energy in an urban area.<br>It could be measured the amount of energy used by studying the urban morphology and topology (high buildings, paved and cemented areas "urban canyon") and exhaust from cars factories, thermal plants.	The paper presents a method to measure the wind and the solar energy flux.<br>Entropy indicator is expressed by the ratio between the entropy variation due to energy losses of buildings and the entropy variation due to the solar energy gain of a mesh area.
(Tambo, 2006) [50]	A distinction between low entropy society and high entropy society. LES has centralized energy (fossil) HES has dispersed energy (solar).<br>Urban areas require low-entropy concentrated energy supply, but for rural, high-entropy application could be tried.<br>(*) low entropy = fossil[1]	City is a system boundary, managed through *"…high-rate consumption of low-entropy, high-quality energy, such as electricity and petroleum, with an integrated reuse system, and running on open material/heat metabolism with the external environment through strictly controlled boundaries."*	Entropy as a characteristic of energy: [high entropy solar energy - - soft energy] vs [low entropy fossil/nuclear energy – hard energy].<br>Gross Domestic Production (GDP) is related to a rate linked to energy. High GDP should consider high efficiency (Japan as example).
(Marchettini et al., 2006) [36]	Entropy variation in open systems is the sum of two terms: external entropy fluxes and internal entropy production. Only irreversible processes produce entropy.<br>The destruction of both biological and cultural diversities, the unique thought and homogenization take inescapably to the thermal death. In the same way a country, a system that makes apolitical dogma of its isolation, of its refusing of cultural contamination.<br>The transition from a low entropy input to high entropy output describes the evolution of a far from equilibrium system as a dissipative structure and the irreversibility of such a pattern according to the Prigogine's concept of arrow of time.	the evolution and maintenance of a city is possible due to natural resources inputs and outputs of heat and wastes (from ecosystems). The appearance of order (of city) is possible due to greater disorder in the environment. [deS = – diS // dS = 0] the disorder is generated in other place, out of the open system. This is a non-equilibrium steady state that should not be confused with thermodynamic equilibrium, and in which order may be created from disorder. The configuration of a complex system, or organization of parts into a whole, is therefore a steady state and depends on interactions among constituents of the system (responsible for the entropy change diS) and perturbations from the external environment (responsible for the entropy change deS). The configuration is always dynamic: it is not static in time but changes whenever external conditions change.	For open system - sum the negative entropy produced inside the system with the positive entropy created in the environment.<br>dS = diS + deS // diS > 0<br>dS increment in the entropy.<br><u>diS - entropy production</u> (heat, disorder) caused by irreversible processes within the system<br>deS - entropy of exchange processes between the system and its environment<br>evolution: deS = – diS // dS = 0<br>Entropy change depends on two processes: interactions among constituents of the system diS and exchanges with the external environment deS.
(Filchakova et al., 2008) [46]	Although the entropy of isolated physicochemical systems tends to evolve irreversibly to equilibrium (dSi > 0), open systems may be maintained in	Cities are complex dynamic entities. open systems, constantly importing and exporting energy and matter across their boundaries; the output invariably being less ordered due	Refers to the 'the entropy pump' concept. The state of a human-modified system is contrasted, by the addition of 'artificial' energy, to that of a natural ecosystem. The

[1] the energy is organized within a system, as reflected in its entropy, a measure of the dispersal of energy across microscale degrees of freedom. Radiant energy is progressively converted to various forms of higher-entropy energy. The low-entropy energy changes entropically as it moves through the Earth system. Radiant energy arriving from the Sun gets converted into chemical, kinetic and thermal energy, stirring myriad entropy-producing flows. The energy ultimately leaves as high-entropy heat waste that radiates back into space. we use energy to support the basic metabolisms of billions of people, based on the consumption of low-entropy food created as a product of farming and photosynthesis (…) <u>we also draw on low-entropy sources of energy — from fossil fuels, nuclear fission, and renewable energy sources</u> — for external purposes such as building, manufacturing, transport, heating, lighting and so on. Our total power consumption in this domain is 10–100 times larger than for metabolism (…) In standard economic analyses, energy in these two broad domains is simply taken as a resource much like any other; entropy is ignored.[67]

	non-equilibrium states. The entropy change dS in such a system is then dS = dSi + dSe.<br>The more energy that is 'pumped' through a system, the greater the degree of organization that can emerge to dissipate this energy.	to irreversible internal processes (dissipation).<br>The paper aims to represent urban metabolism in an operational way and the extent to which thermodynamics can be used to help us quantitatively determine urban sustainability.	model allows calculation of the entropy of a non-equilibrium system under anthropogenic pressure without having to directly calculate the entropy due to individual physical processes. Formulas are given a value as GPP0 (the gross primary production) should be characterized for urban system. Referred from (Svirezhev,2000).
(Tiezzi et al., 2008) [39]	Internal entropy changes due to interactions. External entropy is related with perturbations. The configuration of a complex system, or organization of parts into a whole, depends on interactions among constituents of the system (responsible for the entropy change diS) and perturbations from the external environment (responsible for the entropy change deS).	Cities as dissipative system. "Order out of chaos" Absorption of external input (negentropy) and emission of internal output (entropy, heat) is a principle that works for social systems, economics, human settlements and all their dynamics. Cities absorb flows of high-quality energy from the external environment and emit heat, wastes and pollutants; their internal entropy decreases by self-organization in the form of structures, information, social patterns, and economy.	Resource flows feed these dissipative cities, as if they were ecosystems composed of organisms and are metabolized and continuously used to sustain its ordered structure during the time.<br>Cities are physical systems in contact with various sources and sinks; matter and energy flow through them from the sources to the sinks.
(Browne et al., 2009) [33]	Entropy as waste (high-entropy material). Open, self-organizing systems do not run down in this way is that they are able to import available energy and material from their host environments, which they use to maintain their internal integrity. Such systems also export the resultant entropy in the form of waste and disorder to their hosts.	Useful low-entropy materials enter, undergo a series of changes in their energy and entropy state and, after a time lag, the **residual high-entropy materials or wastes** are dissipated or returned to the environment from various points in the economic process.<br>City-region is an open, complex system, which processes **material flows and inputs and discharges waste outputs**.<br>Policy targets for dematerialization. To measure product and waste flows in an Irish city-region using the principles of metabolism and mass balance [2].	metabolic inefficiency indicator- an empirical indicator to measure resource efficiency, using a ratio between waste disposal and product consumption.<br>In order to complete a material and product flow analysis for the study area and assess urban metabolic inefficiency, it was necessary to quantify (i) production, imports, and exports of raw mate rials; (ii) manufactured good production and trade; and (iii) waste production or generation.
(Fistola, 2010) [51]	Human entropy, referring to increase of negative effects on earth. The energy problem and environmental emergency are strictly correlated entropic cases.	City as one of main entropic systems. Referring to Prigogine, urban systems should be considered as systems with high "anthropic entropy". The target is to reduce anthropic entropy.<br>The concept of *Ecotownplanning*, entails that territory is non-renewable resource.	Not measures but present basic strategies for town planning (orientation, capturing natural energy or rainwater). 3 phases are presented for ecotownplanning: (i) Knowledge, (ii)Decision, (iii) Action. Phase (i) include "Reading, measure and analysis of urban entropy". GIS is used and some base mapping is suggested.
(Fistola, 2011) [52]	Is also about *Ecotownplanning* see previous reference.<br>Entropy as negative effect. High entropy is manifested in the alarming pollution of air and soil but also of water, electromagnetic (OEM) and acoustic.<br>The general crisis is due to the production of entropy in one of the subsystems, which is transmitted, with a ripple effect, to all the others and to the entire city.	The entropic phenomena are therefore related to human activity concentrated in particular in urban contexts.<br>The paper argues that social individualism is one of the main problems and that the first system that needs to change is the socio-anthropic system, referring to behavior of citizens, operators, and governance.	Present the same list than the previous paper (Fistola, 2010).<br>Measures are more general and not directly related to entropy. It is a speculative proposal.

[2] Sector analyzed: (i) food and organic waste; (ii) textiles and leather; (iii) paper and cardboard; (iv) chemicals, rubber, and plastic products; (v) metallic products and other household durable manufactured goods; (vi) industrial machinery and equipment; (vii) transport machinery and equipment; (viii) wood products and furniture; and (ix) construction materials and non-metallic mineral products

(Fistola, 2012) [5]	Paper seeks to identify the characteristics of anthropogenic entropy.<br>Entropy is the phenomenon to be detected and prevented in order to achieve urban sustainability.<br>Entropic phenomena (from pollution to social conflict).<br>Example of the entropic condition is energy dissipation that should not be considered only as the poor use of resources but, for instance, as the waste of food connected with the production of municipal waste[3].<br>[the concept of "dissipation" is central for Fistola, definition of entropy. For the author "dissipated" food becomes entropy].	the main antagonist of city sustainability is anthropogenic entropy.<br>City is experiencing high levels of entropic growth related to locally based human activities. The city is a dissipative structure that absorbs energy and gives off entropy.<br>A city subsystem (human subsystem) can be viewed as an "entropic generator" and can trigger a chain of damage that can lead to the collapse of the entire urban system.<br>Finally, the concept of Entropic Clusters is presented. In an urban system, when system loses its self-organizing capability the parts that are no longer distinguished and separated by the system structure tend to coagulate into entropic clusters which "tend to behave as black holes" producing more entropy.	Entropy can be distinguished in:<br>(i) direct action, expressed as soil use (how, where, for how long) - which is represented by soil use in terms of location, extent, and duration.<br>(ii) entropy-induced action, carried out by the activities producing air, electromagnetic noise, water pollution. This consists in **emissions** affecting the air and water quality air, as well as generating electromagnetic and noise pollution.<br>An example using food waste (from crops, industry, and supermarkets) this waste is "dissipated material" that becomes entropy i.e., waste, which implies a further cost for its disposal, without considering the harmful effects on landfills or incineration systems.
(Gößling-Reisemann, 2011) [41]	An entropy-based measure for resource consumption. in LCA Resource consumption as a loss of potential utility of these resources.<br>The decrease of the system's entropy (by export) is the increase of the environment's entropy (by import) and vice versa. The more irreversible a process, the more potential utility is lost.<br>The change in potential utility inside a system is a negative decreasing function of the entropy change. ⇔ entropy change is equal to internal entropy production. ➔ physical approximation to the loss of potential utility = entropy production.	Not related to cities or urban areas.<br>But it could be linked city to the idea of Technosphere: About LCA - are mainly dealing with flows into and out of the Technosphere but pay little attention to the ways these flows are dealt with within the Technosphere. Although it is true that many of the effects on nature can best be described by measuring the inputs and outputs, the actual solution to environmental problems is most often found within the techno sphere.<br>The basis for an analysis of entropy production in a process is a detailed material and energy balance, including their thermodynamic parameters.<br>Using Umberto LCA Tool, by editing material properties, entropy flows can be visualized in the software.	Entropy production might serve as a proxy for measuring real consumption covering the physical aspect of transformations.<br>Irreversibility of the transformation process reflects the loss of potential utility.<br>$\Delta S_{sys} = \Delta_e S_{sys} + \Delta_i S_{sys}$.<br>*Similar - (Marchettini et al., 2006)<br>The internally produced entropy measures the irreversibility of the transformations taking place inside the system.<br>We can measure $d_e S_{sys}$ if we know all flows of energy and matter into or out of the system at hand, because the exchanged entropy is always the entropy "of something" that is exchanged with the environment, be it materials, heat, or radiation
(Gyftopoulos, 2011) [68]	Entropy is a property in the same sense that energy is a property or momentum is a property.<br>energy change of a system must equal the energy transferred into the system, and that its entropy change must be greater than or at least equal to the entropy transferred into the system.<br>Like energy, entropy can be transferred between systems by means of interactions.	More related to thermodynamics.<br>Not related to cities or urban areas.	Related to thermodynamics.<br>(…) Entropy values are expressed in many different units such as joules per Kelvin (J/K), kilocalorie per Kelvin (kcal/K), and British thermal = unit per Rankine (Btu/R). In particular, it turns out that 1 Btu/lb R 1 kcal/kg K.
(Nemeş et al., 2012) [69]	The paper is about spatial entropy, concept developed by Batty, 1974.<br>Three elements of triple base line were considered to determine the entropy degree of a city *Marghita,	In urban terms, the order can be maintained even if entropy increases, by extending the urban surface.<br>Spatial entropy through Batty's method in a synthesized form was applied to the city of Marghita population, turnover and green area.	The determination of entropy degree was achieving by applying statistical physics function on open system of three elements: population, turnover ($), and green spaces. Thus, applying Batty's developed function for the three analysed elements, the entropy degree was calculated.
(Bodini et al., 2012) [42]	The development capacity (DC), which is the joint entropy of flows, would	Ecosystem model into usable tools to investigate metropolitan areas as integrated	Refers to Odum's 24 attributes to describe ecosystem development. Specifically

[3] It mentioned that "The amount of fruit and vegetables wasted in 2009 could have met the needs of a nation similar to Italy" - The reasons of this dissipation are the shape and/or look of the products or more directly to the picking costs being higher than the market costs.

	express Odom's entropy. DC quantifies the maximum potential that a system has at its disposal to achieve further development, intended at improving organization of flows so that a higher quantity of currency is handled efficiently. This study shows that DC, the proxy for entropy, would be increasing with urban size, contrary to the expectation.	systems. A complementary concept to entropy is stability. Odum hypothesized that stability would augment with ecosystem development. As for entropy, the expectation is that it would be lower in the mature than in the early stages of ecosystem succession.<br>[Hence, entropy increase with urban size.]	refers to entropy (attribute #23) (Odum, E.P., 1969)[4].<br><br>A formula to calculate DC of the system (which it is related to Odum entropy) is presented.<br>$\mathrm{DC} = T_{..}\sum_{i=1}^{N+2}\sum_{j=1}^{N+2}\frac{T_{ij}}{T_{..}}\log_2\left(\frac{T_{ij}}{T_{..}}\right) = \sum_{i=1}^{N+2}\sum_{j=1}^{N+2}T_{ij}\log_2\left(\frac{T_{ij}}{T_{..}}\right)$
(Xuan et al., 2012)<br>[47]	The entropy of the urban ecosystem was defined as a state parameter indicating system disorder, and it could be divided into entropy flow and entropy production.<br>Entropy flow refers to the entropy change resulting from the exchange of material and energy flow between urban socio-economic system and natural support system. includes sustaining input entropy from natural ecological subsystems to socio-economic subsystems and imposed output entropy caused by the cost of natural resources for the socio-economic subsystems.<br>Entropy production pollutants and energy loss generated in the inner operation of socio-economic systems, and as the entropy change resulting from the construction of the ecological environment and infrastructure. Includes destructive metabolism entropy, caused by environmental pollutants with socio-economic activities, and regenerative metabolism entropy, which indicates material energy stored in the urban environmental protection and infrastructure in the urban metabolism process.	Using physical and chemical concepts (e.g., oxidation and reduction) could clearly describe the timeline dynamics characteristics of an urban ecosystem.<br>Considering that urban ecosystems always develop in the direction where entropy has continuously increased (i.e., increasing disorder)<br>An urban ecosystem is a typical dissipative structure deviating from equilibrium, so its entropy flow and entropy production have different meanings from the traditional ones. Urban ecosystem entropy flow can represent the harmonious capacity of system. Entropy production reflects the capacity for system reduction and reproduction in metabolism processes, so it can indicate the vigor of the system.<br>Entropy change, which refers to the overall development of an urban ecosystem, can indicate system order and fitness.<br>[increase order = decrease entropy].<br>urban ecological regulation based on entropy is used to analyze and control entropy flow of the system by means of regulating system structure to reduce the system's internal friction and increasing infrastructure construction and environmental protection.<br>[more time of an urban system, more order, and less entropy].	Entropy as tool to measure disorderliness. an entropy evaluation indicator system for the urban ecosystem was constructed by associating ecosystem characteristics at the town scale. an entropy assessment indicator system was proposed. **[see figureFigure 65]**<br>Combine the regulation indicators values of the urban ecosystem entropy for the planning year 2020 with those of the evaluation time period (2000-2007). After normalization, apply equations (1) and (2) to calculate information entropy of the indicators.<br>**Quantitative model based on information entropy and the harmonious developmental** degree model, for analyze entropy change, the harmonious degree, and the developmental degree of an urban ecosystem.<br>Results: the system will increase order with time, after the regulation program is brought into effect, entropy flow, entropy production, and total entropy will be reduced compared with the evaluation time period, and system order will be significantly enhanced.
(Lai et al., 2013)<br>[70]	Entropy was used to measure the degree of order in decision and spatial structures - following Shannon entropy. It is a simulation using information entropy and is not related to impacts or thermodynamics.	The order brought about by planning is man-made and Euclidean whereas the order generated through self-organization of complex system is natural and fractal. Both types of order can be measured through entropy.	A calculation is presented, using 4 variables (problems, solutions decision makes and places) but it is out of the scope of the research.<br>[It could be revised when dealing with informal settlements from spatial organization perspective].
(Liu et al., 2013)<br>[53]	Waste treatment acts as a balanced system for entropy turbulence.<br>Entropy as disorder: all production (energy transformation) in a city is a dissipative process that converts raw materials in finished products producing entropy (disorder) in the process. all energy transformation processes move towards increasing entropy [paper focus in EMERGY as main methodology – including systemic diagrams with sinks].	urban development as a sink of entropy transfer and as a source of releasing negative effects into the environment.<br>The urban sustainable development is not linear but rather pulses (Odum).<br>natural system in cities is used as an entropy supply pipe to transport negative entropy to a heat sink [city].<br>[entropy is reduced in urban areas but increase in environment – a image about entropy in a systemic model is included].	The level of entropy could measure unsustainability in the economic system. In urban development occurs dissipative process when the system releases materials or energy into the environment, thereby reducing the level of entropy.<br>Hence the socio-environmental damages of the urban system must be quantified.

[4] For Urban metabolism: The largest number of Odum's attributes can be interpreted to imply that mature systems exhibit more cycling and greater internalization of medium (i.e., P/R ratio, #1 in the Odum's list; P/B ratio #2; mineral cycles # 15; nutrient exchange rate # 16; role of detritus in nutrient regeneration # 17; internal symbiosis #20)

Cabral et al., 2013) [7]	Entropy used to describe the structure and behavior of systems.<br>Entropy concept review in urban sprawl studies, seeking to clarify its uses in urban sprawl studies for monitoring and management.<br>Includes a table with initial authors about entropy and urban system.<br>Entropy measures the level of dispersion of an urban area (sprawl) to enable city administration to prevent or mitigate inefficiencies of vulnerability.<br>Entropy as an indicator of performance and risk in urban system.<br>[this paper focus in spatial distribution and urbanization – entropy not directly related to environmental impacts or thermodynamic]	Entropy addresses three big features of urban structure and behavior: the position/location, the mechanic/flow. networks, and system scaling/size.<br>In the case of the paper, it used to measure urban sprawl.<br>Is desirable low entropy in transportation, utilities, services and in the arrangement of built-up areas. However, some redundancy and diversity (entropy) in the urban area is required, so that the city can better resist long-tail events likely to compromise its functioning (natural catastrophes, man-made events).<br>High levels of entropy are very demanding for transport and other infrastructures, whereas low entropy levels increase the risk of social and economic segregation.	Entropy concept has been applied to measure urban sprawl with the integration of remote sensing and geographic information systems.<br>The calculation of Shannon's Entropy, an urban sprawl index using remotely sensed data can efficiently identify and characterize the degree of spatial concentration or dispersion in a specific area.<br>There is a range of values in which entropy could be tolerated without compromising its efficiency and/or its resilience. If entropy falls below HMin, then the urban area is too uniform and, therefore, vulnerable to changes or power laws; if entropy exceeds HMax, then the urban system will be unable to efficiently allocate the necessary resources for the system to function.
(Fistola & La Rocca, 2014) [37]	Speculative proposal of entropy indicators. Entropy is related to negative conditions (urban pollution, energy waste, excessive production of urban waste, increment of vulnerability, high social conflicts, high crime rate, etc.) that may produce adverse effects and discrepancies within the urban system, related to the improper use of available resources [what is "expected" is the sustainability of a system, if the system move in opposite direction is related to entropy].<br>indicators of urban entropy as the opposite to sustainability (positive state "positive indicators") while entropy can be measured by parameters describing negative states or having negative impacts on urban systems.<br>The paper presents a proposal for the entropy indicators (as negative of sustainable indicators)..	Urban entropy is the main obstacle to sustainable planning. First it is needed to identify and measure entropy inside five main urban subsystems. (1) Physical, (2) functional, (3) psycho-perceptive, (4) geomorphologic, (5) anthropic. Each subsystem has static and dynamic components.<br>The paper mentions that problems arise when subsystems change their normal evolution trajectory and start a considerable production of entropy.<br>The study mentions that "it is very difficult to define entropy through suitable indicators describing the state of urban systems".<br>The paper presents the "functional entropy area", a zone where entropy allows the city to evolve, and then the recoverable and unrecoverable zones.	Measuring the change in entropy within a complex system could represent a useful tool to manage it. The "evolution entropy", represents a condition that the system needs in order to evolve, and that the system is able to absorb. When entropy exceeds the boundary of the evolution area, the system risks passing into the unrecoverable entropy area.<br>Entropy ***can be*** considered as the opposite of sustainability – but both are complex phenomena that cannot be easily reduced to a simple reverse relation. For the authors, revisiting sustainability indicators ***could be*** the possible starting point in order to achieve the final target of this study: to assess urban entropy.<br>entropy, intended as a condition of crisis of urban livability, can be useful to measure the level of unsustainability (i.e., entropy state) versus sustainability.
(Bristow & Kennedy, 2015) [29]	Entropy production is related with increase in primary energy use, which occurs when cities grow.<br>The paper is framed within the thermodynamics of the living system and the city as a dissipative structure (Prigogine). Biological systems are highly complex and ordered, far from equilibrium. These ordered structures were created by increasing disorder outside the system. These structures are also present in nonliving phenomena (i.e., thermal convection cells). To maintain these structures requires dissipation of energy (destruction of exergy) [named dissipative structure].	Cities are not on a steady state, and it is not clear if for this type of growing system Maximum Entropy Principles are applied.<br>Energy is dissipated as heat at increasingly higher rates as the cities grow, thereby destroying exergy and increasing the rate of production of entropy. More densely constructed and larger cities have greater intensities of anthropogenic exergy destruction. [then more entropy production]<br>Do cities grow in ways such that they maximize the production of entropy? Might indicate that they do (applying Prigogine) - living systems at first increase their entropy production and energy dissipation rates as they grow, but the rates decrease with maturity Aoki 1995, 2008	The strength of thermodynamics is its ability to describe aggregate properties at macro scale from complex micro scale.<br>The Prigogine equation for a version of the second law of open system was referred.<br>$\frac{dS}{dt} = \frac{dS_I}{dt} + \frac{dS_E}{dt} \quad dS_I = 0$<br>$dS_E = -dS_I \leq 0$<br>dSi is internal and dSe is external.<br>In open system entropy can be maintaed at a steady state dS=0 by interchanging energy with exterior, so, dSe is a negative influx of entropy.<br>*Similar - (Marchettini et al., 2006)
(De Rosa & Salvati, 2016) [71]	Entropy is related with spatial distribution of cities and with	The paper stablishes the case for urban evolution of Naples.	Do not present characterization or measurement with regard entropy and urban studies.

	morphology. Entropic morphology is similar with scattered morphology.	Entropic organization of urban system as a synonym of spontaneity, lack of planning, deregulation, informality, disorder; and opposite to compactness.	
(Lei et al., 2016) [32]	Not directly mentions entropy, but material inputs to the city as negative entropy.<br>Present the concept of anabolic and catabolic[5], mentioning that in cities a tremendous high heat dissipation occurs and after accounting for catabolism, the city retains less than 10% of total input for anabolism.<br>Referring to Prigogine and Odum, systems far from equilibrium can only exist by a continued flow of energy or matter (negative entropy) from outside the system.<br>Mentioned entropy as "negative entropy materials" which is absorbed by cities to support its survival and its development.	City metabolic flows arise from material use, food consumption, and urban development; materials are stored as infrastructure; and materials and wastes are moved through manmade circulatory systems, with pollutants released to the air, land, and water.<br>the city's metabolic rate increases, leading to accelerated consumption of natural resources, losses of farmland and forest, decreases of species diversity, and increased traffic and pollution. Imported anabolic resources are classified in four types of resources (see ~~figure~~Figure 07) [this classification might be use for entropy measure] - [evaluate if heat dissipation, could be relate to entropy – also, the paper evaluates solid and liquid flows and for gases, some analytical methods were adopted].	Cities require inputs of energy and materials, and therefore represent.<br>A dynamic and complex system. so metabolic theory can be used to quantitatively examine these flows.<br>urban metabolism concept is used to quantity the flows of resources (inputs of energy and materials) and of wastes (solid, liquid, and gaseous) through an urban ecosystem.<br>Several metrics can be used to quantify metabolic activities and relationships (anabolism to catabolism ratio, anabolism per capita, anabolism per unit area. Heat dissipation should also be quantified since metabolic process are not perfectly efficient. [Some of these metrics might be related to entropy].
(Coldwell, 2016) [72]	The paper develops the concept of Entropic Citizenship Behavior. Entropy is applied to organizational citizenship behavior. Entropy happens when performance seriously negatively, affecting urban organizational sustainability.	Not related to urban impacts or thermodynamics not related to pollution or energy, more related with social entropy. Aspects as racial segregation are mentioned. Entropy as an indicator of organizational systems sustainability in the urban environment.	Review of methods to evaluate entropy in organizational systems in relation to human organization (social entropy). The paper make reference to Fistola notion of "entropic generator" and highlight the idea that a single subsystem could generate entropy in other system from particular human activities.
(Fistola & La Rocca, 2017) [73]	Two types of urban entropies can be defined: endogenous (related to internal conditions of urban subsystems that might be generated by unplanning) and exogenous (relates to external causes, natural and anthropic).<br>Tourism as one sensitive urban function regarding entropy due to an excess of tourist cause malfunctions and according to authors this is entropy.<br>It is presented the concept of "entropic generator" which it could be a subsystem that generates a chain of damages, reducing its complexity.	Urban sustainability as the opposite of urban entropy, which represents both the "dark side of the urban system" and the negative component of each urban subsystem.<br>The author mentioned that tourism is probably the most emblematic activity that can generate entropy, arguing that when tourist activity becomes too invasive in the city, it generates those entropic processes that progressively lead the city towards a slow degradation.	Metrics of tourism as entropic generator are presented. Some negative impacts of tourism are presented.<br>[For other countries, identify the activity or subsystem that is an entropic generator and study the metrics and data could be the methodology – not always is tourism].
(Patrakeyev et al., 2017) [74]	From an urban metabolism perspective, the paper evaluates the state and efficiency of the urban environment.<br>Entropy is presented as a basic thermodynamic concept than can be used to understand metabolic processes in urban environment.<br>Entropy is defined as a measure of the orderliness of the system, depending on the information content – [a measure of disorder and chaos of a system (definition from thermodynamics)].	In our case, entropy is a measure of the orderliness of the system, depending on the information content.<br>When internal organization of the system is high (ordered structure of the system) means less entropy.<br>A dissipative process is the physical, chemical, and biological process that conduct to an increase in entropy.<br>the quality of the urban environment has been an assessment based on 32 input	To be effectives, systems should minimize the degree of energy dissipation or the degree of energy production.<br>the study measure efficiency of energy use by analyzing energy flows in the UE.<br>[not directly related to entropy and sustainability and urban impacts].

[5] In catabolism, the organism breaks down organic matter and harvests energy by means of cellular respiration (dissipative process); in anabolism, the organism uses energy to construct its components (constructive process) [32]

		indicators covering transport, city-building and social-economic subsystems of the city.	
(Purvis et al., 2017) [24]	**Entropy production is almost solely related to flows of energy, it cannot capture material wastes or pollution, and thus the entropy 'dumped', into the surroundings is really only waste heat.**<br>It is possible to link entropy and sustainability by employing the production of entropy as a measure of urban energy efficiency. The efficiency is applied to processes which use or result in heat. the second law efficiency can be linked to lost work, and thus it can be seen that entropy production and exergy destruction provide measures of the second law efficiency.<br>[If entropy could be measure only in energy, it is possible to account the quantity of energy that will require to restore a system to previous state? If energy and matter are interchangeable, how this could help to identify the exergy from matter?]<br>**Thermodynamics does not include the notion of usefulness or utility, and because of this, failure to capture the human degradation of resources. But is the best tool to measure the utility of a source of energy "but this is as far as it goes" "entropy fails as a measure to quantify the impact of human waste into the environment**.	The author argues that rather than focusing on the entropy produced by urban systems, it is more meaningful to **focus on the urban outputs** that have negative implications for sustainability whether that is anthropogenic heat or pollution, material waste, greenhouse gases. [according to Purvis this pollution, heat, waste, and gases is not entropy, entropy is related with energy].<br>Critiquing Georgescu-Rogen: It can be argued that the utility of the material flow is degraded in the human system but the link to thermodynamic entropy is merely an analogy. To some extent, it can be argued that entropy production can serve as a proxy measure of utility loss, but entropy fails in accounting for qualitative aspects (according to GR) – critiquing also: **low entropy** natural resources enter the economy where they are degraded and transformed into **high entropy** wastes. **low entropy → high entropy.**<br>Critiquing Prigogine: cities produce a large amount of entropy which is dumped into the surrounding environs. This is mostly true, cities use large amounts of energy, so a large production of entropy is inevitable.<br>[Less clear are the consequences of this for the surrounding environment itself].	Thermodynamic entropy as a measure of energy efficiency.<br>The author refers to the fact that entropy production is closely related with the destruction of exergy.<br>$Ex_{destroyed} = T_0 S_{produced}$.<br>Entropy production and exergy destruction are measures of how efficiently energy is used.<br>Entropy production and exergy destruction provide equivalent measures of efficiency means that the two quantities may be used interchangeably given the factor of environmental temperature, T0. However, absolute exergy and entropy are distinct, the exergy of a system can include kinetic and potential energy.<br>Entropy needs to deal with energy flux, and the heat produced by a city is small comparing to the effects of CO_2 for instance.<br>However, the author concludes that "major difficulty of this realization is the inability of entropy to quantify material flows and degradation in a meaningful way"- entropy can't help in the move from linear to circular metabolism.
(Pelorosso et al., 2017) [38]	The role of urban green infrastructure planning in building low-entropy cities is explored. The unused component and space should be limited.<br>During any process, useful energy, also defined as exergy or work capacity, is destroyed and entropy (disorder or waste) is produced.<br>**Entropy exported to the biosphere is due to direct and in direct city wastes**; the former deriving from urban metabolic cycles of socio-ecological and technical systems, the latter produced by unsustainable extraction of resources in source countries and ecosystems to uphold urban systems.	A low entropy city by using Urban Green Infrastructure (UGI). UGI planned to follow the SLT would **increase urban complexity, reducing both the city's internal entropy and its external entropy discharges** (e.g., pollutants, floods, thermal radiation).<br>**[more complexity = less entropy]**<br>Actual cities commonly require and employ high value energy (with a high exergy component, e.g., oil, gas) and release unsustainable, scarcely reusable, low value energy (entropy), which often constitutes an environmental problem and a cause of ecosystem alteration.<br>The paper proposes the concept of low entropy city as a new boundary concept of city, defined as a responsive and conscious autopoietic human sociocultural niche that evolves and grows, enhancing its socio-ecological and structural complexity (reducing internal entropy) by adding and optimizing functional elements and synapses among those elements, while wastes (exported entropy to the biosphere) are minimized.	Use Urban Green Infrastructure (UGI) in unused and underused space [residual, vacant terrains (i.e., urban sprawl) or products of abandonment by industry or residents]. Include buildings, rooftops, walls, streets, squares, and open areas in general and can therefore emerge in a strictly urban context or in a rural/urban fringe (e.g., metropolitan peripheries) or within/around industrial agglomerations.<br>Some new entropy indicators are presented, based on low-entropy city principles, two exemplificative urban evaluations based on these indicators are examined: urban storm water management and social degradation.<br>Some strategies for low entropy cities are the use of renewable and local energies, local food chains, investment in public transport and smart mobility, intelligent management systems for blocks of buildings, heat-cascades (i.e., the multi-stage reuse of residual thermal energy by temperature level), water reuse and so on.
(Pelorosso et al., 2018)	Entropy is a measure of the disorder, or waste of the city, can be considered an	The paper develops the concept of low entropy city. LEC are urban systems with	Some new indicators are used to evaluate the entropy of an urban system in terms of

	indicator of the diversified impacts of the urban development on the biosphere.<br>Entropy exported to the biosphere is represented by direct and indirect city wastes. Direct wastes derive from urban metabolic cycles of socio-ecological and technical systems, indirect wastes are produced by unsustainable extraction of resources in source countries and ecosystems in order to sustain the same urban system.<br>It is worth mentioning that water amount can exceed the overflow weir at the outlet of the study area during critical rainfall events. **At such times, the entropy (waste) is not managed by the treatment plant and is discharged into the sea by the drainage pipe directly**.<br>"Further research is therefore necessary both to define entropy more appropriately in ecological/environmental terms (e.g., impact on urban climate, biodiversity) and to investigate possible implications on social aspects."	lower entropy release into the biosphere by mirroring the natures circular economy ecosystem. LEC aims to investigate what is consumed, how energy and matter are used and where waste might induce socially and environmentally negative significances.<br>LEC in the context of UGI (urban green infrastructure) planning aimed at optimizing the storm water cycle and thus reducing its negative impacts on urban and extra-urban systems, using NBS Natural base solution.<br>Second-law thinking as planning paradigm, aiming to increase exergy and consequently reduce the production of waste (entropy) - [Entropy is related to waste production, and it is related to exergy in a direct relation, more exergy less entropy, maybe the concept of efficiency is connected]<br>Urban areas can increase their socio-ecological structure and complexity (negentropy) only by increasing the disorder and random-ness (entropy) in their host system, the biosphere urban planners should employ free forms of energy, following the low-entropy concept, NBSs should be planned where excessive or unused energy, water and wastes are present.	water quantity and quality parameters. The proposed indicators have an explicit spatial character to allow for practical decisions on urban planning. They are classified in internal entropy indicators and external entropy indicator, that measure the impact of urban system in external areas.<br>The variation in entropy indicator values shows the operational potential of the low-entropy concept for storm water management in urban contexts.<br>All the proposed entropy indicators are derived from the outputs obtained through the implementation of the US-EPA managerial model called Storm Water Management Model (SWMM, release 5.1.012).<br>The proposed internal indicators are Entropy infiltration and Entropy storage. For each the author present equations. For external indicators, overflow, sewage discharge and Total suspended solids. Thru calculations the paper presented results in a spatial reference of the Bari Metropolitan Area.
(Wang, 2018) [44]	Despite evaluates green development the evaluation is based on the entropy method [information entropy, statical, used to calculate the weight of an index]<br><br>[not related to entropy and thermodynamics]	Use of the entropy method to develop the urban green development evaluation index system from five aspects (1) enhancement of living environment, (2) treatment and utilization of pollutant, (3) ecological efficiency improvement, (4) optimization of economic growth (5) development of innovative potential.	The data is from China statistical office. The data was standardized to use in entropy calculations. [as method for top-bottom reference is interesting]<br>Five-aspect considered and the proposed index system and criterion for evaluate green development are proposed.
(Ahmad, 2019) [75]	The concept of entropy is used as a synonym of variance, referring to an increase or a reduction in energy consumption.	Urbanization in small cities increase entropy (variance of energy consumption) while in larger cities the entropy diminishes.	Paper is out of scope.
(Bostanci, 2019) [35]	[one new aspect mentioned was the definition of Gandy of entropy "The growing presence of nature within former industrial landscapes can be conceived as a kind of urban entropy whereby the distinction between human artifice and ecological succession becomes progressively blurred"].	[explanation of how information theory was adapted to urban studies].<br>[mentions many aspects of entropy within urban studies – from efficient use in energy to heat map of city structure].	[the paper is a literature review of previous papers; main point for this study is that the division of entropy studies in two types: physics entropy studies (Pelorosso, Fistola) and information statistical studies].
(Fistola et al., 2019) [76]	Entropy as a negative state, which can affect the urban system in all its components.<br>Entropy is produced when one or more subsystems change the evolution trajectory.<br>Another idea: **an increase in entropy leads to a decrease in available energy and when energy, when energy is no longer available, the system slides into the greatest level of disorder – so entropy is referred as a measure of**	The subsystem division (same author, previous studies) is presented.<br>The new vision of the open complex systems, new aspects to consider: the capacity of adaptation, the presence of chaotic attractor, the dissipative structure, self-organizational features, and the autopoietic ability.<br>Referring to the urban system, entropy can be considered as a condition of scattered unconstructiveness representing an inappropriate use of the economic, social,	Methodologically Entropy is related to the tourist flows traced through data from personal devices.<br>[The application takes the concept of entropy not as central method of evaluation, but as a theoretical reference].

	**disorder**. [key idea for interior exterior entropy],	and territorial resources required for its evolution.	
(Prestamburgo et al., 2019) [77]	In the abstract entropy is mentioned because of lack of rules and planning. Environmental impacts inefficiency and reduction of ecological network are also mentioned, but the relation with entropy is not stablished.	Not related with entropy and thermodynamics.	[out of scope]
(Purvis et al., 2019) [9]	For the thermodynamic entropy (the one that is developed in the present research) the authors refer that Rees & Wackernagel (1997) describes cities as "entropic black holes", which draw in large amounts of entropy and matter, and "export the resultant entropy (waste and disorder)" to maintain their "highly ordered dissipative structure." [entropy is defined as waste and disorder]. However, anything described as a source of 'disorder' such as pollution cannot be simply coded as 'entropic' or acting to increase entropy in the thermodynamic sense.<br>Critical view of the use of entropy from a qualitative perspective. Entropy is not a direct measure of 'utility' or the lack of. In its thermodynamic sense, is not a sufficient measure of the negative externalities caused by human activity.	From statistical entropy: entropy is maximal when probabilities are evenly distributed, and zero when concentrated in a single location, and it was used for representing a measure of spatial concentration or dispersion. Using this definition, indices were used to measured: ethnic diversity, urban sprawl, segregation, diversity of urban land use, and the geographic distribution of species (biodiversity). In this type of studies, GIS and remote sensing are usually applied. Also, it has been used to modeling the pattern of transport flows within an urban system or welfare' in the allocation of housing.<br>For thermodynamic entropy, the author mentions that this area of thermodynamics is still an active work in progress and lacks an established corpus.<br>It is argued that the ordered dissipative structure of the city is maintained at the expense of "increasing entropy or disorder in the environment", citing trends of natural capital depletion, pollution emissions, and other adverse anthropogenic ecological consequences.<br>A paper about the Metaphor of Entropy as Disorder is referred (Haglund, 2017)	Using Oxford dictionary definition, the paper also proposes three interrelated entropy definitions used in urban context are presented: the thermodynamic, the figurative, and the information statistical. In contrast to Bostanci, Purvis divided the "physical" entropy in thermodynamic and figurative.<br>**The physical definition of entropy** in the cities has its roots with both the work on non-equilibrium thermodynamics of Ilya Prigogine, and that of the economist Nicolas Georgescu-Roegen, who considered entropy in relation to the economic process. It is often, however, presented **qualitatively** and can border on the second more figurative definition of entropy.<br>According to Purvis et al, there is a wider confusion that pervades applications of thermodynamic entropy, and how the distinction between its figurative use as an analogy, and proposed operationalization as a well-define physical quantity, is not always clear.<br>To be of any use as an indicator one must be able to measure thermodynamic entropy in thermodynamic units. Without a mathematical formulation, it remains a metaphor, that according to the author fails to be useful, sowing confusion rather than bringing clarity.
(Luqman & Al-Ansari, 2020) [78]	The paper is a thermodynamic analysis of an Energy-Water-Food applied to coastal communities.<br>There is not a clear definition of entropy withing the paper, but from the equation that are using it is referring to the thermodynamic entropy, where S (entropy) is:<br>S: Specific entropy (kJ/kg.K)[6] [kilojoule per kilogram per kelvin]	The paper is more technical and specific (not analysing urban flows or pollution) but it could be an interesting approach on proposing new systems that could reduce entropy (some complex equations are presented to calculate thermodynamic models) for instance, part of the heat produced is use for food drying. The system has been analysed by development of thermodynamic model of the complete system.	The paper proposes a new multigeneration system, which subsystem consist in a Biomass combustor, Concentrated Solar Power (CSP), a Rankine Cycle, a desalination unit, and an Absorption Cooling System (ACS).<br>For calculations a software was used Engineering Equation Solver (EES) software [this tool is specific to an engineering system].
(Y. Wang et al., 2020) [79]	The concept of **ecological load entropy** is proposed as a tool to evaluate ecological functional vulnerability of natural ecosystems.<br>Heterogeneity in the load distribution of system components makes the system highly vulnerable to cascading failure >	[One interesting approach is the developing of the **vulnerability** concept as an analytical tool, especially for natural ecosystem – here the "disturbance or stress" to which a system is exposed could be used to measure the entropy, but in a reverse way, i.e., how to return the system to the non-disturbance condition].	Data is obtained from government agencies from China. >> Land system with a higher ecological load entropy has a lower heterogeneity and is more robust and less vulnerable to disturbances (e.g., rapid urbanization).<br>An important review of "ecological services" is stablished, mentioning that

[6] kilojoule per kilogram per kelvin is a decimal multiple of the SI (International System of Units) derived unit of specific heat capacity, joule per kilogram per kelvin - material has a heat capacity of 1 J/kg·K if heat energy of one joule is required to raise the temperature of one kilogram of this material by one kelvin.

	and load entropy is used to measure networks heterogeneity.<br>A system with a **higher load entropy has a lower heterogeneity** and is more robust and less vulnerable to disturbances. Then > Entropy is related with robustness. High entropy, low heterogeneity, high order, high robustness, and low vulnerability.	[The more heterogenous the system is the low entropy it has].	usually this are often considered public goods, common pools resources and not integrated into format markets and underestimated.
(Netto et al., 2020) [45]	Using information entropy to evaluate social interactions in relation to urban spatial configuration.<br>Entropy is mentioned as a measure of **probability** in the face of uncertainty (Shannon, 1948), and a measure of **disorder** (Prigogine and Stengers, 1984). Hence paper main hypothesis is: If entropy relates to trends toward **uncertainty and disorder**, and if social systems face entropy all the time, continuity, and self-maintenance (low-entropy state) become significant issues. Uncertainty triggers entropy. The reduction of entropy brings together issues of sustainability of social and urban systems.	Social actions = information / Information level of dispersion = entropy<br><br>By coordination, cities can reduce the entropy of actions that might otherwise disperse and lead to social instability and fragmentation.<br><br>City as a space for coordination - Solutions to global problems strains high levels of coordination, massive numbers of people will need to change their habits and focus their actions on sustainable directions.	An agent-based model (ABM) considering agents and places. Coordination to convert numerous potential actions into smaller sets of actual interactions.<br>**[City as a mean of communication and communication as a specific form of energy]**. Energy exchanged between social entities in an urban context takes a rather specific form: communication, the transmission of information as meaningful content that is mutually understandable by people interacting.<br>Entropy is used to assess how successful social and urban systems can be in dealing with their own self-maintenance and sustainability.
(Fistola et al., 2020) [43]	In the study entropy is defined as: entropy (or the production of energy that is no longer available) is considered as **a widespread negative condition of the urban system**, which produces negative effects and malfunctions within the system itself. However, some paragraph later they mentioned: As regards the scope of this study and considering the definition of entropy based on Shannon's information theory (1948) and Bayesian inference (…) it is possible to refer to entropy as a **subjective property of a system linked to the amount of information available for the system itself**. The amount of uncertain in information is related to disorder.	Urban Vulnerability is correlated with entropy. Urban risk = V x E x P (risk, vulnerability, exposure, probability)<br>Is about internal entropy. urban vulnerability as an expression of systemic entropy. Entropy is produced due to incorrect use of social, economic, geographical, and territorial resources in urban subsystems.<br>City as a complex system = complexity = aleatorily = chaos >>> Entropy as a measure of chaos.	Is about assessing internal entropy.<br>Three central urban system entropy should be measured: the physical, functional, and social systems. Considering sustainability as the opposite of entropy. Use sustainability indicators. Negative indicators could measure entropy.<br>Entropy is generated during any transformation process, where useful energy is lost and irrecoverable and generates entropy in form of disorder and waste. This the definition from SLT, and the link with transformation and energy – the positive energy lost is exergy.
Following section is not part of the results or the literature review but where include as references of different perspectives regarding entropy.			
(Pelorosso, Gobattoni, Ripa, et al., 2018) [34]	low-entropy UGI planning<br>the strategy emphasizes the role of modelling in the assessment phase and identifies several entropy indicators able to be easily applied by planners.	Urban green infrastructure (UGI).	Entropy and urban metabolism are presented in order to investigate the main applications of, and gaps in, urban spatial planning.
(Ho, 2013) [80]	There is a world of difference between circular and linear input-out. It is also the difference between zero-entropy circular economy and the dominant economy of infinite competitive growth and maximum dissipation (entropy generation).	Circular Thermodynamics of Organisms and Sustainable Systems.	Thermodynamic efficiency requires that ΔS approaches 0 (least dissipation) and $\Delta H = 0$; or ΔG approaches 0 via entropy-enthalpy compensation, i.e., entropy and enthalpy changes cancelling each other out.
(Tan et al., 2019) [81]	comparing exergy and mass use for Production, Manufacturing, Construction, Wholesale & Retail, Transport & Storage, Accommodation &	Flows by sector, mainly exergy and mass.	Cities as open system networks. Metrics for (1) effectiveness of utilization and (2) conversion

	food, Information & Communication, Finance & Insurance, Business activities, Domestic, and other services		Ecological Network Analysis (ENA) as the main methodology
(Nazarnia et al., 2019) [82]	Shannon's entropy is not a reliable method for measuring urban sprawl and produces misleading results. An alternative suggestion could be the metrics of "Weighted Urban Proliferation" (WUP) and "Urban Permeation" (UP).	Urban sprawl.	Six case studies from Canada (including Montréal) investigate the sensitivity of entropy to variations in the size and number of zones. Geospatial data and ArcGIS 10 were used for the measurement of entropy.
(Fan et al., 2019) [83]	gravity model and information entropy were introduced for the study of the spatial network structure of an urban system.	The entropy of Urban System Network	Entropy theory to analyze the spatiotemporal pattern of urban network evolution in efficiency and quality – Measures entropy according to interaction between city nodes [regional level]. Is more about communication between cities.
(Algaze, 2018) [84]	Energy in ancient cities is related with population. More population more energy and less entropy. In contemporary cities, the use or injection of energy as a negentropic flow.	Population input to counteract entropy in ancient cities.	Entropy is not measure but it could be argued that population is a form energy, so population as energy will also create entropy.
(Asprilla et al., 2018) [85]	The concept of entropy is related to the disorder of the city in relation to transportation system and mobility in the city.	Entropy of cities related to structural organization, related to chaos, lack of organization or disorder.	Measure mobility.
(Chen & Huang, 2018) [86]	Not clearly stablished. Could be argued that spatial concentration could be linked to entropy.	Spatial measures.	Spatial entropy. Study the conversion of entropy into fractal dimension for spatial analysis.
(Boeing, 2019) [87]	Regular urban grid is identified as "high ordered". Lima is part of the study.	Measure spatial networks (urban grid) complexity.	To calculate use the Shannon entropy of the city's orientations' distribution (Shannon, 1948) used to calculate the entropy of the unweighted/simplified street orientations
(Braham et al., 2019) [88]	It could be possible to measure the entropy of high emergy city zones.<br>[It could be argued that the more emergy the more entropy produced.]	Each category of land-use in a census track. Three main categories: agriculture and forest, non-residential and residential.	Method of emergy synthesis to understand the interaction of natural and socioeconomic factors an accounting method, it mostly uses Unit Emergy Values (UEV) inputs multiplying them by the flows of energy, materials, and services in a system to quantify the total amounts The UEVs data used in the study were drawn from many sources.
(Tong et al., 2021) [89]	Entropy as a measure to evaluate the recyclability of waste. It could be used for Waste flow in cities.	Municipal Solid Waste (MSW) in China.	Entropy-weighted recyclability index (EWRI) is developed to quantify the recyclability.
